# Multi-omics analysis reveals distinct non-reversion mechanisms of PARPi resistance in BRCA1- versus BRCA2-deficient mammary tumors

**DOI:** 10.1016/j.celrep.2023.112538

**Published:** 2023-05-19

**Authors:** Jinhyuk Bhin, Mariana Paes Dias, Ewa Gogola, Frank Rolfs, Sander R. Piersma, Roebi de Bruijn, Julian R. de Ruiter, Bram van den Broek, Alexandra A. Duarte, Wendy Sol, Ingrid van der Heijden, Christina Andronikou, Taina S. Kaiponen, Lara Bakker, Cor Lieftink, Ben Morris, Roderick L. Beijersbergen, Marieke van de Ven, Connie R. Jimenez, Lodewyk F.A. Wessels, Sven Rottenberg, Jos Jonkers

**Affiliations:** 1 Division of Molecular Pathology, Oncode Institute, the Netherlands Cancer Institute, 1066CX Amsterdam, the Netherlands; 2 Division of Molecular Carcinogenesis, Oncode Institute, the Netherlands Cancer Institute, 1066CX Amsterdam, the Netherlands; 3 Department of Biomedical System Informatics, Gangnam Severance Hospital, Yonsei University College of Medicine, Seoul 03722, Republic of Korea; 4 OncoProteomics Laboratory, Department Medical Oncology, Amsterdam UMC, 1081HV Amsterdam, the Netherlands; 5 Division of Cell Biology, Oncode Institute, the Netherlands Cancer Institute, 1066CX Amsterdam, the Netherlands; 6 Cancer Therapy Resistance Cluster and Bern Center for Precision Medicine, Department for Biomedical Research, University of Bern, 3088 Bern, Switzerland; 7 Institute of Animal Pathology, Vetsuisse Faculty, University of Bern, 3012 Bern, Switzerland; 8 Mouse Clinic for Cancer and Aging, Preclinical Intervention Unit, the Netherlands Cancer Institute, 1066CX Amsterdam, the Netherlands

**Keywords:** breast cancer, homologous recombination, BRCA1, BRCA2, PARP inhibitor, therapy resistance, multi-omics

## Abstract

BRCA1 and BRCA2 both function in DNA double-strand break repair by homologous recombination (HR). Due to their HR defect, BRCA1/2-deficient cancers are sensitive to poly(ADP-ribose) polymerase inhibitors (PARPis), but they eventually acquire resistance. Preclinical studies yielded several PARPi resistance mechanisms that do not involve BRCA1/2 reactivation, but their relevance in the clinic remains elusive. To investigate which BRCA1/2-independent mechanisms drive spontaneous resistance *in vivo*, we combine molecular profiling with functional analysis of HR of matched PARPi-naive and PARPi-resistant mouse mammary tumors harboring large intragenic deletions that prevent reactivation of BRCA1/2. We observe restoration of HR in 62% of PARPi-resistant BRCA1-deficient tumors but none in the PARPi-resistant BRCA2-deficient tumors. Moreover, we find that 53BP1 loss is the prevalent resistance mechanism in HR-proficient BRCA1-deficient tumors, whereas resistance in BRCA2-deficient tumors is mainly induced by PARG loss. Furthermore, combined multi-omics analysis identifies additional genes and pathways potentially involved in modulating PARPi response.

## Highlights

• HR recovery drives PARPi resistance in BRCA1-KO tumors but not in BRCA2-KO tumors
• HR recovery in PARPi-resistant BRCA1-KO tumors occurs mostly via 53BP1 loss
• HR-deficient PARPi-resistant BRCA1-KO tumors show increased immune infiltration
• PARG loss is the most common alteration in PARPi-resistant BRCA2-deficient tumors

## Graphical abstract

**Figure:**
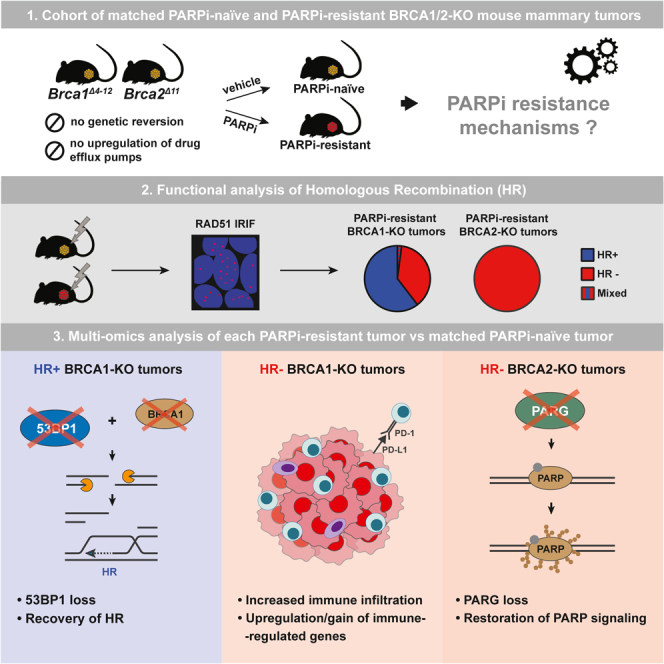

## Teaser

Bhin et al. use multi-omics and HR analysis of matched treatment-naive and PARPi-resistant BRCA1/2-KO mouse tumors to investigate non-reversion mechanisms driving spontaneous resistance *in vivo*. HR restoration via 53BP1 loss and restoration of PARP signaling via PARG loss are the two dominant resistance mechanisms in BRCA1-KO and BRCA2-KO tumors, respectively.

## Introduction

The observation that many oncogenic events render cancer cells reliant on specific and druggable biological pathways is a premise of targeted therapies for personalized cancer treatment. Unfortunately, the selective pressure that initially kills cancer cells is also a driving force in selecting cells that acquired drug resistance. A better understanding of the recurrent molecular patterns of resistance in specific genetic contexts is therefore instrumental to improving clinical outcomes.

One example of cancer dependencies that can be exploited therapeutically is the defect in the repair of DNA double-strand breaks (DSBs) through homologous recombination (HR) due to BRCA1 or BRCA2 inactivation.^1^^,^^2^ Unlike the other DSB repair pathways, HR enables the accurate repair of DNA lesions, as it uses the newly replicated sister chromatid as a template. Both BRCA1 and BRCA2 are essential in this process. While BRCA1 is required for the initiation of HR by promoting the end resection of the DSB, BRCA2 acts further downstream and, together with PALB2, stimulates the recruitment of RAD51 recombinase to the resected single-stranded DNA.^3^ The HR defect resulting from the loss of BRCA1/2 can be targeted through the inhibition of poly(ADP-ribose) polymerase (PARP) enzymes PARP1 and PARP2.^1^^,^^2^ PARP1/2 have been implicated in several DNA damage response (DDR) pathways, including the repair of DNA single-strand breaks (SSBs) and DSBs and the stabilization of replication forks (RFs).^4^ Catalytic inhibition as well as trapping of PARP1/2 on the DNA by PARP inhibitors (PARPis) leads to replication-coupled DSB formation, which in turn requires HR for error-free repair.^5^ BRCA1/2-defective cells can only employ error-prone repair to resolve the DSBs caused by PARPi treatment, resulting in the accumulation of chromosomal aberrations and cell death by mitotic catastrophe.^6^ The success of this approach resulted in the clinical approval of four different PARPis for the treatment of several types of cancers with HR defects.^7^

Despite the clinical benefit, sustained antitumor responses to PARPis are often hampered by the emergence of resistance. Previous studies have delineated several mechanisms by which BRCA1/2-deficient tumors evade PARPi toxicity.^7^ Independently of HR, PARPi resistance may be induced through (1) cellular extrusion of PARPis by upregulation of the drug efflux transporter P-glycoprotein (Pgp) encoded by *Mdr1a/b*;^8^ (2) partial restoration of catalytic PARP activity through loss of poly(ADP-ribose) glycohydrolase (PARG);^9^ (3) PARP1 downregulation/inactivation as well as mutations that abolish PARP1 trapping^5^^,^^10^^,^^11^; and (iv) restoration of RF stability.^12^^,^^13^^,^^14^ All these mechanisms result in PARPi resistance by limiting PARPi-induced DNA damage rather than restoring the capacity of BRCA1/2-deficient cells to efficiently repair DSBs. In contrast, HR restoration as a result of secondary (epi)genetic events that lead to reactivation of functional BRCA1/2 may fully cancel the initial susceptibility to PARPis. In addition, genetic screens and *in vivo* studies in preclinical models demonstrated that inactivation of the 53BP1-RIF1-shieldin DSB end-protection pathway, which inhibits HR and is antagonized by BRCA1 during S phase, partially restores HR and confers PARPi resistance in BRCA1-deficient cells.^15^^,^^16^^,^^17^^,^^18^^,^^19^^,^^20^^,^^21^^,^^22^^,^^23^^,^^24^^,^^25^^,^^26^^,^^27^^,^^28^^,^^29^^,^^30^

While multiple mechanisms of acquired PARPi resistance have been reported in preclinical *in vitro* models, their clinical relevance remains unclear. To date, the best clinically documented mechanism of resistance is the restoration of BRCA1/2 function by secondary (epi)genetic events (e.g., reversion mutations).^31^ However, these results might be biased by the fact that PARPis were initially approved for second-line maintenance therapy following first-line treatment with platinum-based chemotherapies. Since (epi)genetic reactivation of BRCA1/2 function has been shown to be the main mechanism of platinum resistance in *BRCA1/2*-mutated tumors, it is plausible that some of these patients might have already developed BRCA-proficient, and therefore PARPi-resistant, tumor clones as a result of a first-line treatment.^32^^,^^33^^,^^34^^,^^35^ Moreover, reactivation of BRCA1/2 function is not found in all patients with refractory tumors,^33^^,^^36^ suggesting that BRCA1/2-independent PARPi resistance is relevant in the clinic.

The PARPis olaparib and niraparib have recently been approved as first-line maintenance therapies, and clinical trials have started to test PARPis as single-agent neoadjuvant therapy.^37^ With more patients likely to receive PARPis earlier in the course of disease, it is important to understand what are the most frequent mechanisms of acquired PARPi resistance, and if these differ between *BRCA1-* and *BRCA2*-mutated tumors, in order to predict PARPi response and to develop strategies to overcome resistance. In the absence of available clinical data, we sought to answer these questions by combining functional analysis of HR status with molecular profiling of a collection of matched PARPi-naive and PARPi-resistant mouse mammary tumors that harbor large intragenic deletions of *Brca1* or *Brca2* genes that cannot be spontaneously restored. Overall, our study shows that functional differences between BRCA1 and BRCA2 in the repair of DSBs also impact the resistance patterns in PARPi-treated tumors. While HR restoration accounted for the majority of BRCA1-deficient tumors, it did not occur in BRCA2-deficient tumors, suggesting that HR cannot be restored in *Brca2*-mutated tumors that cannot undergo BRCA2 reactivation. Moreover, among the previously reported resistance mechanisms, loss of 53BP1 and loss of PARG were the most dominant alterations in PARPi-resistant BRCA1-deficient and BRCA2-deficient tumors, respectively. Dysregulation of other known resistance factors was only sporadically observed, suggesting 53BP1 and PARG as potential biomarkers of acquired PARPi resistance. Additionally, our analysis yielded a list of potential genes and pathways involved in PARPi response and provides evidence that tumor-intrinsic alterations in pathways regulating the tumor microenvironment may influence PARPi efficacy.

## Results

### HR restoration drives PARPi resistance in BRCA1-deficient tumors

**Figure 1:**
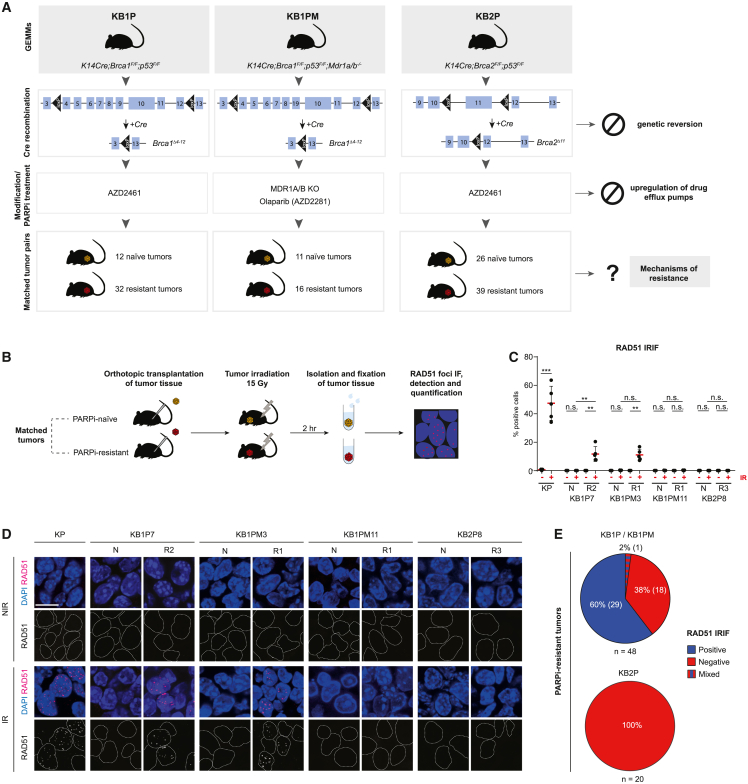
HR restoration drives PARPi resistance in BRCA1-deficient tumors (A) Outline of the generation of matched PARPi-naive and PARPi-resistant KB1P(M) and KB2P tumors and of the experimental approach. (B) Schematic representation of the RAD51 IRIF formation assay. Cryopreserved PARPi-naive and PARPi-resistant tumors were orthotopically transplanted into syngeneic recipient mice, and upon outgrowth to 500 mm3, DNA damage was inflicted by locally applied ionizing radiation (IR) at a dose of 15 Gy. 2 h post-irradiation, tumors were isolated, and fixed tissues were used for RAD51 immunofluorescence imaging. (C and D) Quantification (C) and representative images (D) of the RAD51 IRIFs for the different matched KB1P(M) and KB2P tumor pairs and control KP tumors; IR, irradiated; NIR, non-irradiated; scale bar, 10 μm; data in (C) represented as percentages of positive cells (≥5 foci/nucleus) per imaged area (single data point, typically 100–200 cells/area). n = 5 per imaged area. Data are represented as mean ± standard deviation (SD); ^∗∗∗∗^p < 0.0001, ^∗∗∗^p < 0.001, ^∗∗^p < 0.01 (two-tailed Mann-Whitney U test, followed by Benjamini-Hochberg [BH] correction). (E) Pie charts showing the outcome of the RAD51 IRIF assay in PARPi-resistant KB1P(M) and KB2P tumor cohorts; percentages and numbers of individual tumors analyzed are indicated; n, total number of individual tumors analyzed from the indicated models. p = 0.0001 (two-tailed Fisher’s exact test).

To study the contribution of BRCA1/2-independent PARPi resistance mechanisms in BRCA1/2-deficient tumors, we used two genetically engineered mouse models (GEMMs) of BRCA1-associated breast cancer, *K14cre;Brca1*^*F/F*^*;Trp53* ^*F/F*^ (KB1P) and *K14cre;Brca1*^*F/F*^*;Trp53* ^*F/F*^*;Mdr1a/b*^−/−^ (KB1PM), as well as a GEMM of BRCA2-associated breast cancer, *K14cre;Brca2*^*F/F*^*;Trp53* ^*F/F*^ (KB2P)^8^^,^^9^^,^^38^^,^^39^^,^^40^ (Figure 1A). In these models, long-term treatment of mammary tumors with PARPis leads to spontaneous induction of resistance, which is preserved upon tumor passaging.^38^^,^^41^ Importantly, the tumors arising in these models harbor large intragenic deletions in the *Brca1* or *Brca2* genes,^39^^,^^40^ and thus resistance to PARPis cannot be acquired via reactivation of BRCA1/2 function. Moreover, we eliminated the possibility of Pgp-mediated resistance to the PARPi olaparib by either the genetic inactivation of Pgp (*Mdr1a/b*) in the KB1PM model or by treating Pgp-proficient KB1P and KB2P tumors with the PARPi AZD2461, which is a poor substrate for this transporter.^38^^,^^42^ In total, we obtained 12 PARPi-naive and 32 PARPi-resistant KB1P tumors, 11 PARPi-naive and 16 PARPi-resistant KB1PM tumors, and 26 PARPi-naive and 39 PARPi-resistant KB2P tumors (Figure 1A).

HR deficiency is the basis for sensitivity to PARPis, and thus we hypothesized that HR restoration is the most likely way for tumors to acquire resistance to PARPis. We therefore assessed the HR status of matched PARPi-naive and PARPi-resistant tumors by measuring their capacity to form ionizing radiation-induced RAD51 foci (RAD51 IRIF)^17^^,^^43^ (Figure 1B). As a positive control for this assay, we used an HR-proficient mammary tumor derived from the *K14cre;Trp53* ^*F/F*^ (KP) model and observed the highest accumulation of RAD51 foci 2 h after induction of DNA damage (Figures S1A–S1D). Of note, all tumors exhibited high growth rates prior to irradiation, suggesting that differences in cell cycle distribution between the samples are negligible (Figures S1E and S1F). As expected, we did not detect RAD51 IRIF formation in all PARPi-naive KB1P (12), KB1PM (11), and KB2P (15) tumors tested (Figures S1G, 1C, and 1D), confirming that the *Brca1/2* deletions induced in our models completely abolish HR-mediated repair. Consistent with this, whole-exome sequencing data of PARPi-resistant tumors showed significantly low read coverages of the deleted *Brca1/2* exons in PARPi-naive and -resistant KB1P(M) and KB2P tumors compared with normal spleen samples with wild-type *Brca1/2*, demonstrating Cre-mediated deletion of the *Brca1/2* exons flanked by *loxP* sites (Figures S1H and S1I). Of note, there are two KB1P tumors with relatively high read coverage of the deleted *Brca1* exons, but this is most likely due to high stromal cell infiltration in those two tumors (Figure S1J). Therefore, we concluded that no emerging resistance could be attributed to the selection of clones that retained wild-type BRCA1/2.

A similar proportion of RAD51-IRIF-positive tumors between PARPi-resistant KB1P and KB1PM tumors suggest that these two models developed resistance in a similar manner, which is further supported by indistinctive molecular features between the two models (Figures S2A–S2C). Analysis of the 48 PARPi-resistant BRCA1-deficient (32 KB1P and 16 KB1PM tumors) tumors revealed that 60% (29/48) of the tumors had restored the capacity to form RAD51 foci, including one tumor with a mixed pattern (RAD51-IRIF-positive and -negative areas) (Figure 1E). These results indicate that HR recovery is the predominant mechanism of PARPi resistance in the KB1P(M) models, albeit not the only one. In contrast, none of the 20 PARPi-resistant BRCA2-deficient tumors exhibited RAD51 IRIF^9^ (Figure 1E). Given that PARPi treatment is a potent trigger of HR restoration in the KB1P(M) models, the lack of RAD51 IRIF in the BRCA2-deficient cohort strongly indicates that BRCA2 is indispensable for HR repair.

### Alterations in previously reported PARPi resistance factors

To understand how prolonged PARPi treatment reshapes BRCA1/2-deficient tumors, we performed whole-exome sequencing (WE-seq), low-coverage whole-genome sequencing (LCWG-seq), and RNA sequencing (RNA-seq) on the collection of matched PARPi-naive and PARPi-resistant KB1P(M) and KB2P tumors and identified alterations specific to each resistant tumor compared with the matched naive tumor.

**Figure 2:**
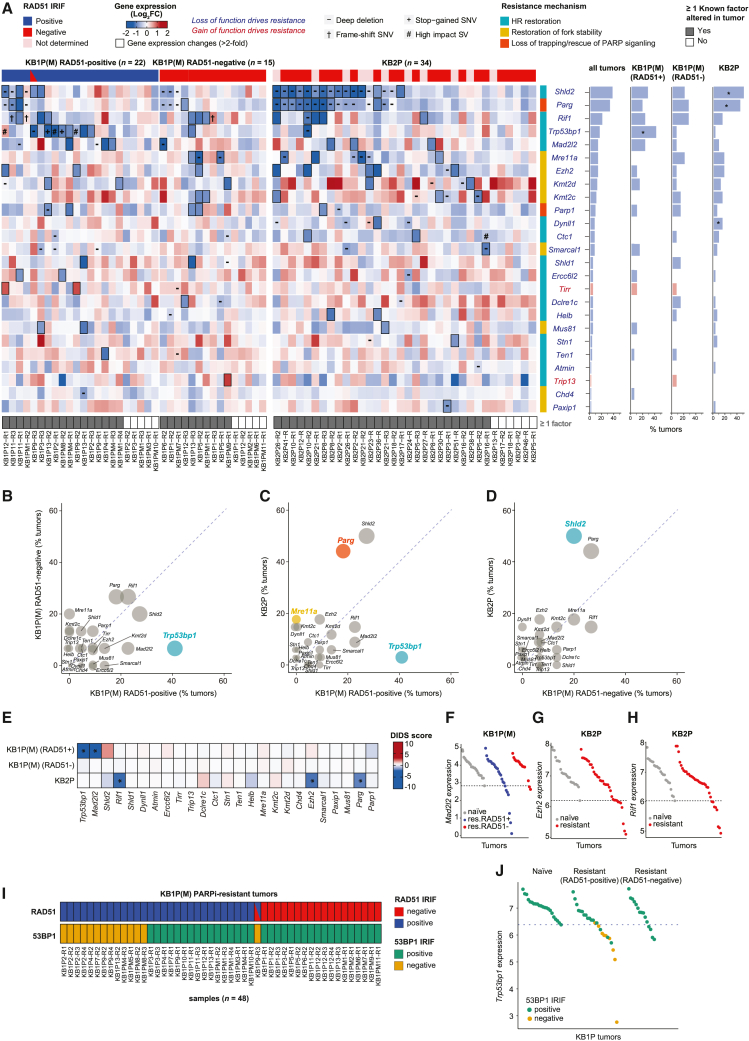
Alterations in previously reported PARPi resistance factors (A) Heatmap (left) of gene expression changes between matched resistant versus naive KB1P(M) and KB2P tumors for the previously reported PARPi resistance genes.^7^ Genes for which loss or gain of function have been reported to drive resistance are indicated in blue or red, respectively. In the heatmap, genomic alterations are marked by different symbols (-: copy-number deletion; +: stop-gained SNV; †: frameshift SNV; #: loss-of-function SV). The resistant tumors with transcriptional alterations (lower or higher than 2-fold compared with matched naive tumors for each loss- or gain-of-function driver) are marked by thicker borderlines. The top panel of the heatmap indicates RAD51 IRIF status, which is a proxy for HR status (positive: blue; negative: red; tumors with both positive and negative areas: blue and red; tumors for which RAD51 IRIF was not determined even though it was expected to be negative: pink). The bottom panel of the heatmap indicates the tumors with alterations in at least one known gene. Resistance mechanisms associated with each gene are categorized by different color bars (light green: HR restoration; purple: restoration of fork stability; light blue: PARP signaling). Frequencies for dysregulation of each gene (by either genomic or transcriptional alterations) are shown in the bar plots next to the heatmap (right). The genes preferentially altered in specific tumor types were assessed by the Fisher’s exact test (^∗^p < 0.05). (B–D) Scatterplots comparing the alteration frequency of each PARPi resistance factor in the different resistant tumor types. The size of the circle is proportional to the sum of the alteration frequency of the two resistant tumor types compared, and circles are colored if statistically significant (Fisher’s exact test, p < 0.05). The color of the circle indicates the resistance mechanism associated with each resistance factor as mentioned in (A). (E) DIDS outlier scores computed from gene expression data for known resistance factors. Red (positive score) and blue (negative score) indicate upregulation and downregulation of each factor in a subset of resistant tumors compared with naive tumors. The genes with significant DIDS scores are marked with an asterisk (permutation-based exact test, p < 0.05). (F–H) Dot plots of *Mad2l2* gene expression in KB1P(M) tumors (F) and *Ezh2* (G) and *Rif1* (H) gene expressions in KB2P tumors where significant DIDS scores were detected. (I) RAD51 and 53BP1 IRIF status in KB1P(M) PARPi-resistant tumors measured by *in situ* IRIF assay. (J) Dot plots of *Trp53bp1* gene expression in KB1P(M) tumors with 53BP1 IRIF status.

We first interrogated if we could find genetic and transcriptional alterations in factors previously associated with PARPi resistance (Figure 2A). To this end, we selected 25 genes reported to drive PARPi resistance due to BRCA-independent HR restoration, restoration of fork stability, or modulation of PARP signaling/trapping^7^ (Table S1). We examined alterations in these genes in the different PARPi-resistant tumor groups with informed HR status, i.e., (1) RAD51-positive KB1P(M), (2) RAD51-negative KB1P(M), and (3) KB2P tumors. Of the 25 genes, 23 have been reported to drive PARPi resistance upon loss of function, whereas 2 genes drive resistance as a result of gain of function. Globally, we found alterations in all known factors analyzed, which occurred at different frequencies in the different PARPi-resistant tumor groups. Nearly 80% (55/71) of all resistant tumors harbored deleterious mutations, copy-number variations, and/or gene expression changes in at least one known factor. *Shld2*, *Parg*, *Rif1*, *Trp53bp1*, *Mad2l2* (*Rev7*), *Ezh2*, *Mre11a*, *Kmt2c* (*Mll3*), and *Kmt2d* (*Mll4*) were among the most frequently altered genes (≥10% of all tumors) (Figure 2A).

We next asked whether certain genes were preferentially altered within the three different PARPi-resistant tumor groups. We found that *Trp53bp1*, one of the best-studied PARPi-resistance factors involved in HR restoration via loss of DSB end protection, was specifically altered in RAD51-positive KB1P(M) tumors (41%) compared with RAD51-negative KB1P(M) (7%) and KB2P (3%) tumors (Figures 2A–2D). In contrast, *Shld2* and *Parg* were preferentially altered by copy-number loss in KB2P tumors compared with KB1P(M) tumors (Figures 2A and 2C). Previously, we reported that loss of PARG causes PARPi resistance independently of BRCA1/2 by restoring PAR formation and restoring the recruitment of DNA repair factors downstream of PARP1.^9^ While we expected *Parg* to be equally altered in both KB1P(M) and KB2P tumors, we found that *Parg* was more frequently lost in KB2P tumors (44%) (Figures 2A and 2C) than in RAD51-positive (18%) and RAD51-negative KB1P(M) tumors (27%) (Figure 2B). The genomic location of *Shld2* is proximal to *Parg* (chr14qB), and thus copy-number loss of *Parg* is often accompanied by concomitant loss of *Shld2* (Figure 2A). Moreover, depletion of SHLD2 has been reported to drive PARPi resistance via HR restoration in BRCA1-deficient cells but not in BRCA2-deficient cells. Hence, loss of *Shld2* in KB2P tumors is most likely a consequence of *Parg* copy-number loss rather than driving resistance to PARPi. In support of this, CRISPR-Cas9-mediated inactivation of SHLD2 did not drive resistance in cultured KB2P tumor cells (Figures S2D and S2E). *Mre11a* downregulation or copy loss was only found in KB2P and RAD51-negative KB1P(M) tumors, suggesting that *Mre11a* is specifically lost in RAD51-negative PARPi-resistant tumors (Figures 2A–2D). This is in line with its key role in HR and suggests that loss of MRE11 may induce PARPi resistance by promoting RF protection.

We also applied detection of imbalanced differential signal (DIDS) analysis specifically designed for the detection of subgroup markers in heterogeneous populations.^44^ This tool is particularly useful for identifying drug resistance factors in a tumor group with multiple resistance mechanisms by detecting genes with outlying expression in the subset of resistant tumors compared with all naive tumors.^9^^,^^45^ DIDS analysis additionally identified *Mad2l2* to be significantly downregulated in RAD51-positive PARPi-resistant KB1P(M) tumors, which is consistent with its role in the 53BP1-RIF1-shieldin pathway involved in DSB end protection and with the observation that MAD2L2 loss promotes HR and PARPi resistance in BRCA1-deficient cells^17^^,^^18^^,^^24^^,^^25^^,^^26^ (Figures 2E and 2F). *Ezh2*, which was previously shown to promote fork stability and PARPi resistance when depleted, was also identified by DIDS analysis to be downregulated in PARPi-resistant KB2P tumors, in accordance with the previous findings that loss of EZH2 impairs response to PARPi in BRCA2-deficient cells but not in BRCA1-deficient cells^46^ (Figures 2E and 2G). Surprisingly, *Rif1* was significantly downregulated in PARPi-resistant KB2P tumors but not in KB1P tumors, suggesting that RIF1 might have 53BP1-RIF1-shieldin-independent functions that could drive resistance in BRCA2-deficient tumors (Figures 2E and 2H). However, CRISPR-Cas9-mediated inactivation of RIF1 did not reduce PARPi sensitivity in cultured KB2P tumor cells, suggesting that RIF1 loss might only drive resistance *in vivo* or that it is a consequence of PARPi treatment rather than causal to resistance (Figures S2F and S2G).

To confirm that PARPi resistance mediated by 53BP1 loss is enriched in RAD51-positive PARPi-resistant tumors, we evaluated the functional impairment of 53BP1 in KB1P(M) and KB2P tumors by analyzing 53BP1-IRIF formation (Figures S2H and S2I). We found that loss of 53BP1-IRIF was only detected in RAD51-positive or mixed PARPi-resistant KB1P(M) tumors, whereas PARPi-resistant KB2P tumors as well as PARPi-naive KB1P(M) or KB2P tumors did not show loss of 53BP1-IRIF (Figures 2I and S1G). Consistently, tumors with loss of 53BP1-IRIF showed lower *Trp53bp1* expression levels than other tumors, suggesting good concordance between the omics data and the functional assay (Figure 2J). Altogether, our analysis revealed multiple known factors significantly altered in RAD51-positive KB1P(M) and in KB2P tumors; however, none were found to specifically explain PARPi resistance in RAD51-negative PARPi-resistant KB1P(M) tumors.

### HR-deficient PARPi-resistant BRCA1-KO tumors show increased immune cell infiltration

We then systematically characterized the three resistant tumor groups beyond the known resistance factors using our genomic and transcriptomics data. To identify recurrent focal genomic alterations between resistant and matched naive tumors, we performed copy-number analysis using RUBIC.^47^ The majority of the significantly recurrent alterations identified in PARPi-resistant tumors were focal deletions (Figures S3A–S3C), including loss of the regions encoding *Mad2l2* in RAD51-positive KB1P(M) tumors and *Parg* in KB2P tumors, respectively (Figures S3A and S3C). In PARPi-resistant RAD51-negative KB1P(M) tumors, we could not identify recurrent focal events, with the exception of one amplified locus on chromosome 8 (Figure S3B). Several genes encoded within the recurrently amplified/deleted loci in PARPi-resistant KB1P(M) and KB2P tumors are associated with DDR pathways (Figure S3D). Gene set analysis (GSA) demonstrated that genes involved in cell cycle and cell division were depleted in PARPi-resistant RAD51-positive KB1P(M) tumors, whereas genes involved in positive regulation of immune cell activation were amplified in PARPi-resistant RAD51-negative KB1P(M) tumors (Figures S3E–S3G). In PARPi-resistant KB2P tumors, we found loss of genes involved in metabolic processes such as phospholipid and DNA metabolism.

**Figure 3:**
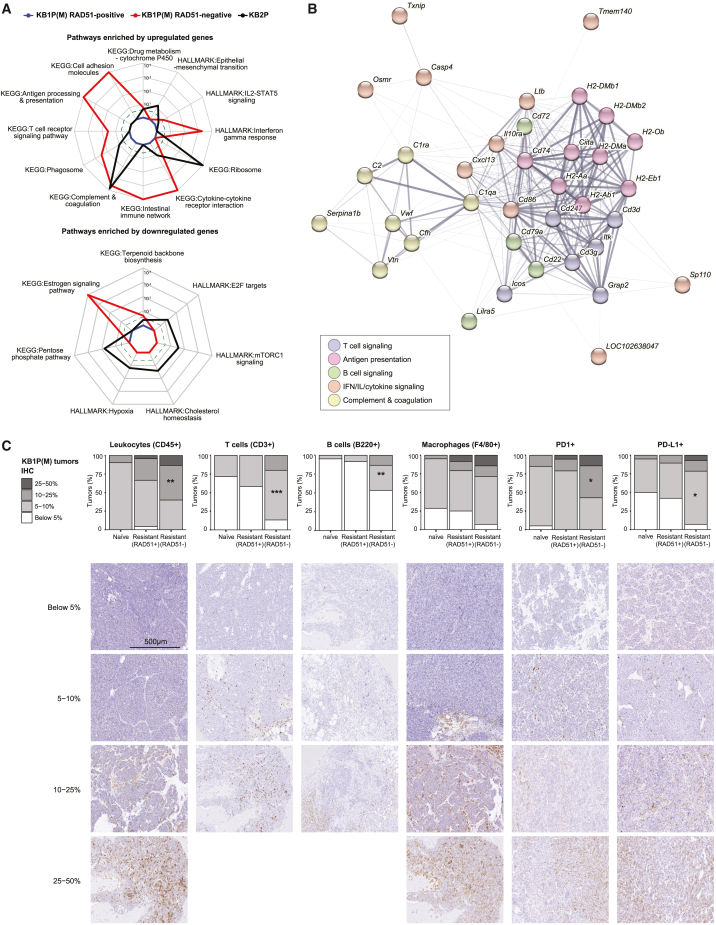
HR-deficient PARPi-resistant BRCA1-KO tumors show increased immune cell infiltration (A) Radar chart showing pathways enriched by upregulated and downregulated genes in KB1P(M) and KB2P resistant tumors compared with naive tumors based on gene expression data. Gene sets from MSigDB Hallmark^50^ and KEGG^51^ were used for these enrichment analyses. The scale of the axis is represented by false discovery rate (FDR; Fisher’s exact test followed by BH correction). The dotted line indicates an adjusted p value <0.25. (B) Co-functionality network constructed by STRING^49^ for the immune-related genes that are significantly upregulated in RAD51-negative KB1P(M) resistant tumors compared with naive tumors. The genes in the network were annotated by MSigDB Hallmark^50^ and KEGG^51^ and colored depending on the annotated pathways. (C) Quantification and representative images of IHC analysis of markers for different immune cells including leukocytes (CD45), T cells (CD3), B cells (B220), macrophages (F4/80), PD1, and PD-L1 in KB1P(M) tumors. Fisher’s exact test was performed to compare the number of samples with IHC staining levels below or above 5% and 10% between naive and RAD51-positive or RAD51-negative KB1P(M) resistant tumors. Asterisks denote the statistically significant enrichment based on the sample grouping by IHC staining level 5% or 10%.

Next, we transcriptionally characterized the different PARPi-resistant tumor groups. Differential gene expression analysis by limma^48^ between resistant and naive tumors identified 26, 349, and 1,058 genes in PARPi-resistant RAD51-positive KB1P(M), RAD51-negative KB1P(M), and KB2P tumors, respectively, including downregulation of *Trp53bp1*, *Parg*, and *Shld2* (Figures S3H–S3J). No known resistance-associated factors were found to be differentially expressed in RAD51-negative KB1P(M) tumors (Figure S3I). GSA of the differentially expressed genes (DEGs) in each group of resistant tumors identified distinct sets of pathways. Interestingly, PARPi-resistant RAD51-negative KB1P(M) tumors showed upregulation of immune and interferon-related pathways, such as antigen processing and presentation, T cell receptor signaling, phagosome, interferon-gamma response, and interleukin-2 (IL-2)-STAT5 pathways (Figure 3A), which might be associated with the enrichment of immune cell regulators with focal amplification in these tumors (Figure S3F). Co-functionality network analysis of upregulated immune-associated genes using the STRING database^49^ revealed several immune cell modules, such as T cells (e.g., *Cd3d*, *Cd3g*, *Cd247*), B cells (e.g., *Cd22*, *Cd72*, *Cd79a*), antigen presentation (e.g., *Cd74*, *Ciita*, *H2-Ob, H2-Aa*, *H2-Ab1*, *H2-Eb1*, *H2-DMa*, *H2-Dmb1*, *H2-Dmb2*), and interferon (IFN) signaling (e.g., IL10RA, CD86, CASP4), suggesting an increase in immune cell infiltration (Figure 3B). We validated these findings by carrying out immunohistochemical (IHC) analysis of markers of leukocytes (CD45), T cells (CD3), B cells (B220), macrophages (F4/80), and PD1- and PD-L1-positive cells, revealing increased expression of all these markers in PARPi-resistant KB1P(M) tumors when compared with naive tumors, with RAD51-negative KB1P(M) tumors displaying a stronger increase compared with RAD51-positive KB1P(M) tumors (Figure 3C). Therefore, our findings suggest that RAD51-negative PARPi-resistant KB1P(M) tumors have higher immune infiltration compared to PARPi-naive and RAD51-positive PARPi-resistant KB1P(M) tumors. We could not observe differences in mutation and copy-number variation loads between RAD51-positive and RAD51-negative PARPi-resistant KB1P(M) tumors, indicating that immune infiltration in RAD51-negative PARPi-resistant KB1P(M) tumors may not be triggered by increased genomic instability (Figures S3K and S3L). Altogether, these data demonstrate that PARPi-resistant tumors display distinct genomic and transcriptomic features depending on BRCA1/2 loss and HR status.

### Multi-omics analysis identifies potential PARPi-resistance factors/pathways

**Figure 4:**
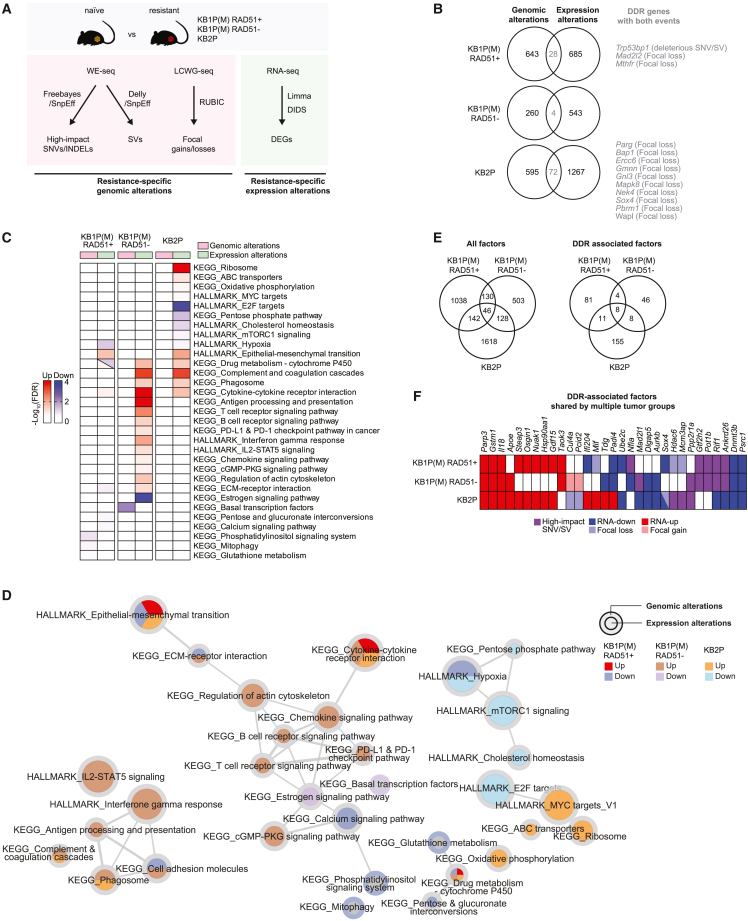
Multi-omics analysis identifies potential PARPi resistance factors/pathways (A) Schematic overview of the analysis to identify resistance-specific genomic and transcriptional alterations from WE-seq, LCWG-seq, and RNA-seq data. For each PARPi-naive and -resistant RAD51-positive and RAD51-negative KB1P(M) and KB2P tumor, (1) deleterious SNVs, indels, SVs, and focal amplifications/deletions (resistance-specific genomic alterations) and (2) DEG sets identified by either limma or DIDS analysis (resistance-specific transcriptional alterations) were selected. DEG sets were extended to capture the genes with both homogeneous (by limma) and heterogeneous behavior (by DIDS) between naive and resistant tumors. (B) Venn diagrams showing the overlaps between the genes having resistance-specific genomic alterations (SNVs, indels, SVs, and copy-number focal gains and losses) and transcriptional alterations (DEGs) in each resistant tumor type. (C) Heatmap of pathways significantly enriched by the genes with resistance-specific genomic and transcriptional alterations. Enrichment, -log_10_(FDR) computed by Fisher’s exact test followed by BH correction, by upregulated and downregulated genes are represented by red and green in the heatmap, respectively. Gene sets from MSigDB Hallmark^50^ and KEGG^51^ were used for these enrichment analyses. (D) Pathway enrichment map for genes with resistance-specific genomic and transcriptional alterations constructed by EnrichmentMap.^52^ Node size represents the size (number of genes) of each gene set, and edges represent mutual overlaps between the gene sets (minimum similarity score 0.3). Node and border colors represent enrichment by the genes with resistance-specific transcriptional and genomic alterations, respectively. (E) Venn diagrams showing the overlaps of the genes having resistance-specific alterations with either genomic or transcriptional alterations across the three resistant tumor types. (F) List of DDR-associated genes with resistance-specific alterations that were identified in multiple resistant tumor types.

To catalog potential PARPi resistance factors, we identified genes displaying resistance-specific genomic (single nucleotide variants [SNVs], insertions or deletions [indels], structural variants [SVs], focal amplifications/deletions) and transcriptional (DEG sets) alterations in each group of resistant tumors by integrating WE-seq, LCWG-seq, and RNA-seq data (Figure 4A). Of note, we extended DEG sets to capture the genes with both homogeneous (by limma) and heterogeneous behavior (by DIDS) between naive and resistant tumors. Overall, we observed limited overlap between genomic and transcriptional alterations across all tumor groups, including several genes involved in DDR (Figure 4B; Table S2). Moreover, the overlap between genomic and transcriptional alterations was mostly derived from copy-number alterations, with the exception of *Trp53bp1*, in which we found truncating SNVs and deleterious SVs leading to nonsense-mediated decay. The limited overlap between genomic and transcriptional alterations indicates that genomic alterations do not always lead to gene expression changes. Nonetheless, several known resistance factors including *Trp53bp1*, *Mad2l2*, and *Parg* displayed both genomic and transcriptional alterations. We next explored biological pathways represented by the identified genes with resistance-specific genomic and transcriptional alterations (Figures 4C and 4D; Table S3). As described before, we found transcriptional upregulation of immune-associated pathways specifically in RAD51-negative KB1P(M) tumors, including upregulation of antigen processing and presentation and B and T cell receptor signaling. (Figures 4C and 4D). In line with the observed increase in PD1- and PD-L1-positive cells in RAD51-negative PARPi-resistant KB1P(M) tumors (Figure 3C), we also found upregulation of immune checkpoint pathways. Interestingly, pathways associated with cell adhesion molecules and extracellular matrix (ECM)-receptor interaction were transcriptionally downregulated in RAD51-positive KB1P(M) tumors but upregulated in RAD51-negative KB1P(M) tumors (Figures 4C and 4D). Moreover, ribosome-associated pathways were upregulated in KB2P tumors. At the genomic level, loss-of-function alterations (mainly copy-number loss or deleterious SNVs and SVs) were observed in the phosphatidylinositol signaling pathway in RAD51-positive KB1P(M) tumors and in pathways associated with basal transcription factors in RAD51-negative KB1P(M) tumors (Figures 4C and 4D). In line with the limited overlap between genomic and transcriptional alterations (Figure 4B), pathways enriched by genes with genomic alterations showed no overlap with those enriched in transcriptional alterations (Figure 4C). Nonetheless, we found 46 genes that carried either genomic or transcriptional alterations in all three resistant tumor groups, including 8 genes (*Parp3*, *Gstm1*, *Il18*, *Padi4*, *Dnmt3b*, *Psrc1*, *Rif1*, and *Ankrd26*) involved in DDR (Figures 4E and 4F). Taken together, integration of genomics and transcriptomics data from PARPi-resistant versus PARPi-naive tumors allowed us to catalog genes and pathways potentially involved in modulating PARPi response.

To prioritize candidate resistance drivers, we identified genes with genomic alterations that had a significant impact on transcriptional changes in the protein-protein interaction network by carrying out driver analysis with DriverNet.^53^ We identified drivers in 25% of genes with resistance-specific mutations and in 23% of genes with copy-number alterations (Figure S4A). Interestingly, DDR genes were more enriched in drivers compared with non-drivers in all PARPi-resistant tumor groups, with the exception of drivers that were derived from copy-number alterations in RAD51-negative KB1P(M) tumors (Figures S4B–S4D), supporting the previous findings that DDR pathways are strongly involved in governing PARPi response.^7^

### *In vitro* loss-of-function screens fail to validate candidate drivers of *in vivo* PARPi resistance

To identify causative drivers among the genes associated with PARPi resistance in KB1P(M) tumors, we performed functional genetic enrichment screens in human BRCA1-deficient cell lines. To obtain a comprehensive set of genes to be functionally tested in the screens, we generated global proteomics and phosphoproteomics data and identified proteins (DE-Prot) and phosphoproteins (DE-Phos) that were differentially expressed between naive and resistant tumors. We combined the DE-Prot and DE-Phos with genes identified from WE-seq, LCWG-seq, and RNA-seq analysis for all KB1P(M) tumors, including tumors whose RAD51 IRIF status was not determined, resulting in 3,727 resistance-associated genes (Figure S4E). Plausible drivers were prioritized by recurrences in multiple datasets, associations with DDR, and potential as network drivers, yielding a final list of 891 putative PARPi resistance driving factors including 53BP1, MAD2L2, HELB, and PARG (Figure S4F).

We then generated a focused lentiviral short hairpin RNA (shRNA) library targeting the identified 891 candidate genes plus 133 non-essential genes as controls (Table S4). Given the strong effect of 53BP1 loss on PARPi resistance, we decided to exclude *Trp53bp1*-targeting shRNAs from the library to prevent them from obscuring less-dominant hits. We introduced the lentiviral shRNA library in the human BRCA1-deficient cell lines SUM149PT and RPE1-hTERT-*BRCA1*^−/−^*;TP53*^−/−^, which were subsequently selected with olaparib for 3 weeks (Figure S4G). MAD2L2 behaved as a positive control and was identified as the top hit in both cell lines, but no other common hits were found (Figures S4H and S4I). We identified RBMS1 as a hit in the screen carried out in SUM149PT cells (Figure S4H), but shRNA-mediated depletion of RBMS1 *in vivo* did not significantly affect the survival of KB1P tumor-bearing mice (Figure S4J). Overall, these results suggest that our *in vitro* loss-of-function screens were insufficient to validate the candidate drivers of *in vivo* PARPi resistance identified from the multi-omics analysis of PARPi-resistant tumors.

## Discussion

In this study, we combined functional analysis of HR status with multi-omics analysis of a collection of matched PARPi-naive and PARPi-resistant BRCA1-knockout (KO) and BRCA2-KO mouse mammary tumors to classify the contribution of previously reported non-reversion resistance mechanisms in a preclinical “*in vivo* reality” of spontaneously acquired resistance. Overall, our analysis highlights the differences in resistance patterns between BRCA1- and BRCA2-deficient tumors and identifies HR restoration via loss of 53BP1 and restoration of PARP signaling via loss of PARG as the two dominant non-reversion resistance mechanisms in BRCA1- and BRCA2-deficient tumors, respectively. Additionally, our analysis generated a catalog of candidate genes and pathways potentially involved in modulating the PARPi response. Expanding the use of PARPis in the clinic should soon provide clinical specimens that will allow us to verify the relevance of our findings.

### Functional differences between BRCA1 and BRCA2 impact PARPi resistance patterns

In our study, we also used PARPi resistance as a tool to probe for different activities of BRCA1 and BRCA2 in DNA repair. BRCA1 and BRCA2 are often mentioned together, partly owing to their tumor-suppressor activities and roles in HR repair. From a biological standpoint, however, BRCA1 and BRCA2 are not functionally redundant in HR. The epistatic relationship between BRCA1 and BRCA2 was first put forward in the context of embryonic lethality by Ludwig et al. almost 20 years ago.^54^ Consistent with this relationship, previous work from our laboratory demonstrated that concomitant tissue-specific deletion of the *Brca1* and *Brca2* genes (KB1B2P) resulted in similar tumor development as single-gene KOs (KB1P and KB2P).^55^ Our present analysis of PARPi resistance mechanisms reveals a clear functional distinction between BRCA1 and BRCA2 in DNA repair. HR deficiency and PARPi sensitivity of BRCA1-KO tumors could be largely reversed by inactivation of the 53BP1-RIF1-shieldin pathway, indicating that BRCA1 plays a role upstream of end resection where it competes with the 53BP1-RIF1-shieldin pathway. In contrast, BRCA2-KO tumors completely failed to restore HR activity, as measured by RAD51 foci formation, underlying the essential role of BRCA2 in RAD51 loading during the HR process. Moreover, we found that the levels of the RAD51 IRIF in the PARPi-resistant KB1P(M) tumors were significantly lower than in BRCA1/2-proficient controls, indicating partial restoration of HR activity in these samples (Figure 1C). This is consistent with previous DR-GFP-based HR reporter assays we performed in 53BP1- or MAD2L2-depleted KB1P cells.^17^ In addition, recent studies have suggested that whereas BRCA1 is dispensable for DNA end resection, its interaction with PALB2 and the resulting promotion of RAD51 loading cannot be fully compensated.^56^^,^^57^ It is therefore conceivable that inactivation of the 53BP1-RIF1-shieldin pathway in BRCA1-KO tumors rescues the DNA end-resection defect but fails to fully restore HR repair due to lack of BRCA1 activity downstream of resection. Altogether, these data show that BRCA1 and BRCA2 have distinct activities in the repair of DSBs by HR and that these differences impact the resistance patterns in PARPi-treated tumors.

### Resistance mechanisms in HR-restored BRCA1-KO tumors

Loss of 53BP1 was the most frequent alteration in RAD51-positive PARPi-resistant KB1P(M) tumors, whereas all other DSB end-protection factors seemed to be only sporadic. In line with this, mice bearing *Shld1/2*- or *Ctc1*-mutated KB1P tumors exhibit only partial response to olaparib compared with mice bearing unmodified KB1P tumors, whereas mice bearing *Trp53bp1*-mutated tumors did not respond to PARPi treatment, resulting in survival curves identical to vehicle-treated mice.^25^^,^^58^ Moreover, loss of 53BP1 has also been observed in patient-derived tumor xenograft (PDX) models with acquired resistance to PARPis, and mutations in *TP53BP1* have been reported in tumor biopsies from patients with metastatic BRCA1-associated breast cancer receiving platinum chemotherapy or PARPis.^27^^,^^59^^,^^60^ Altogether, our results indicate that BRCA1-independent HR restoration driven by inactivation of 53BP1 may be the most common mechanism of PARPi resistance in patients with *BRCA1*-mutated tumors that do not undergo BRCA1 reactivation and that 53BP1 may be a potential biomarker of PARPi response in *BRCA1*-mutated tumors.

### Resistance mechanisms in HR-deficient BRCA1-KO tumors

More than one-third of all PARPi-resistant KB1P(M) tumors were RAD51 negative, indicating that they had not restored HR. Despite the fact that alterations in previously reported resistance factors were found sporadically in PARPi-resistant RAD51-negative KB1P(M) tumors, none of these factors were found to be significantly altered in any of the analyses carried out in this study. Moreover, alterations in these factors did not occur more frequently in PARPi-resistant RAD51-negative KB1P(M) tumors compared with PARPi-resistant RAD51-positive KB1P(M) or KB2P tumors. Nevertheless, GSA of the DEGs identified in PARPi-resistant RAD51-negative KB1P(M) tumors yielded an enrichment in positive regulation of immune response that was in line with the increase in immune infiltration detected by IHC analysis. This suggests that immune-regulated mechanisms might contribute to PARPi resistance in these tumors. In line with this, organoids derived from one of the PARPi-resistant RAD51-negative KB1P(M) tumors (KB1PM7) failed to recapitulate PARPi resistance *in vitro* but upheld PARPi resistance *in vivo*, suggesting that PARPi resistance in this tumor may be driven via cell-extrinsic processes that can only be recapitulated *in vivo*.^61^ Moreover, these tumors preserve PARPi resistance following orthotopic transplantation of tumor fragments into syngeneic mice, indicating that the resistance phenotype is tumor intrinsic.

Previous studies have reported that *BRCA1/2*-mutated tumors display increased immune infiltration upon treatment with PARPi, suggesting that immune infiltration is required for PARPi antitumor efficacy.^62^ In this study, we observed an increase in immune infiltration specifically in PARPi-resistant RAD51-negative KB1P(M) tumors. Of note, we did not observe higher mutation or copy-number burden in these tumors compared with KB1P(M) RAD51-positive tumors (Figures S3K and S3L). However, we found upregulation of genes associated with IFN-gamma response and IL-2-STAT5 signaling pathways (Figures 3A and 3B), which could indicate activation of cGAS-STING signaling. cGAS-STING signaling has been previously reported to be induced by PARPis and results in immune cell activation, which could justify the increase in immune infiltration observed.^62^^,^^63^^,^^64^ Moreover, among the different pathways upregulated in PARPi-resistant RAD51-negative KB1P(M) tumors, we observed upregulation of the PD-L1/PD-1 pathway by GSA (Figure 4C) and IHC (Figure 3C), which is in line with the previously reported upregulation of PD-L1 in response to PARPi treatment and suggests that patients with PARPi-resistant, HR-deficient *BRCA1*-mutated tumors might benefit from PARPi and immune-checkpoint inhibitor combination.^62^^,^^63^^,^^64^^,^^65^ Altogether, these data highlight the role of the tumor immune microenvironment in the response to PARPi.

### Resistance mechanisms in BRCA2-KO tumors

PARG loss was the most frequent alteration in PARPi-resistant KB2P tumors and occurred significantly more often in KB2P tumors than in the other tumor groups, even though a few resistant KB1P(M) tumors carried copy-number loss and/or downregulation of *Parg*. The strong selection for PARG loss in KB2P tumors could result from the impossibility of HR restoration in these tumors. Even so, PARG loss was not more frequent in PARPi-resistant HR-negative KB1P(M) tumors compared with HR-positive KB1P(M) tumors.

Perturbations that occurred more frequently in PARPi-resistant BRCA2-KO tumors than in BRCA1-KO tumors were alterations associated with loss of PARP trapping or rescue of PARP signaling (44% in KB2P versus 32% in KB1P(M); mostly involving *Parg*) and alterations associated with restoration of RF stability (56% in KB2P versus 35% in KB1P(M); mostly involving sporadic alterations). Unlike PARG, perturbations in PARP1 were anecdotal in both KB1P and KB2P tumors, suggesting that PARP1 activity is critical for the viability of BRCA1/2-deficient cells.

In our PARPi-resistant mouse tumors, we observed concomitant loss of *Parg* and *Shld2* through focal deletion of chromosome 14. Although the distance between *PARG* and *SHLD2* is longer in the human genome (37 versus 2 MB), both genes are located on chromosome 10q, and recurrent arm-level deletions in this region have been reported in multiple cancer types, such as lymphomas, glioblastoma, and lung cancer.^66^^,^^67^^,^^68^^,^^69^^,^^70^

Interestingly, GSA also identified upregulation of genes involved in oxidative phosphorylation and ribosome-associated pathways in PARPi-resistant KB2P tumors, which is in line with previous reports that OXPHOS and NAD+ availability can modulate PARPi response.^71^^,^^72^^,^^73^ For example, reduction of intracellular levels of the PARP substrate NAD^+^ via inhibition of nicotinamide phosphoribosyltransferase (NAMPT) or high levels of the NAD^+^ derivative NADP^+^ reduce PARP activity and thereby enhance sensitivity of cancer cells to PARPis.^71^^,^^72^ Moreover, HR-deficient tumor cells show high OXPHOS because they rely on oxidative metabolism to supply NAD^+^ and ATP for PARP-mediated DNA repair.^72^

Overall, our data show that PARPi resistance cannot be achieved via HR restoration in BRCA2-deficient tumors that cannot undergo BRCA2 reactivation. Moreover, loss of PARG is the single most recurrent driver of acquired resistance in BRCA2-KO tumors, yielding PARG as a potential predictive marker of PARPi response in patients with *BRCA2*-mutated tumors.

### Limitations of this study

Our tumor collection recapitulates BRCA1/2 loss-driven tumor formation and acquired PARPi resistance but does not capture the full complexity of the human cancer (e.g., metastatic disease, heterogeneity, hypomorphic mutations). Moreover, human genes do not always have mouse orthologs or play the same functions across the two species. For example, the previously reported PARPi resistance factor SLFN11^13^ does not have a mouse ortholog and that is why it was not included in our analysis.

This study relies on the comparison with naive tumors but does not include the comparison with responsive tumors (before developing resistance). Such a tumor cohort would allow us to identify gene/pathways altered during PARPi treatment before the tumors develop resistance. Additionally, the multi-omics analysis carried out in this study does not include methylome data, and thus we might miss epigenetic mechanisms of PARPi resistance. Nevertheless, changes in DNA methylation are predicted to affect transcriptional activity and should therefore be visible in the RNA-seq data.

In this study, we carried out *in vitro* loss-of-function screens in two human cell lines to validate candidate drivers of *in vivo* PARPi resistance; however, this approach failed to yield common hits other than the already-known factor *Mad2l2*. A limitation of this approach is that loss-of-function screens, such as shRNA-based screens, can only functionally validate candidate loss-of-function factors, which represent 40% (356/891) of all candidate drivers. Additionally, PARPi resistance in tumors without alterations in known resistance genes could be driven by the additive effect of multiple alterations rather than a single event. Resistance could also be driven by genes that modulate the tumor microenvironment, which cannot be adequately modeled in *in vitro* screens. The use of human cell lines in our screens could have also compromised the validation of the candidates identified with our multi-omics approach. Functional screens in KB1P mouse mammary tumor cells could help to understand if our findings are mouse specific.

## STAR★Methods

### Key resources table

**Table:** 

REAGENT or RESOURCE	SOURCE	IDENTIFIER
**Antibodies**		

Rabbit polyclonal anti-RAD51	kind gift from R. Kanaar, Erasmus MC, Rotterdam	N/A
Rabbit polyclonal anti-53BP1	Abcam	Cat#ab21083; RRID: AB_302845
Goat polyclonal anti-rabbit, Alexa Fluor® 658-conjugated	Thermo Fisher Scientific	Cat#A11011; RRID: AB_143157
Goat polyclonal anti-RIF1	Santa Cruz Biotechnology	Cat#sc-55979; RRID:AB_2126818
Rabbit anti-goat, immunoglobulins/HRP	Dako	Cat#P0449; RRID:AB_2617143
Rabbit polyclonal anti-CD45 antibody	Abcam	Cat#ab10558; RRID:AB_442810
Rabbit monoclonal anti-CD3 (Clone SP7)	Thermo Fisher Scientific	Cat#RM-9107; RRID:AB_149924
Rat monoclonal anti-CD45R (B220)	BD Pharmingen	Cat#557390; RRID:AB_396673
Rabbit monoclonal anti-F4/80 (D2S9R) XP	Cell Signaling	Cat#70076; RRID:AB_2799771
Rabbit monoclonal anti-PD-L1 (D5V3B)	Cell Signaling	Cat#64988S; RRID:AB_960250
Rabbit polyclonal anti-PD1	Proteintech	Cat#18106-1-AP; RRID:AB_10732952
Goat polyclonal anti-rat-Biotynated	SouthernBiotech	Cat# 3052-08; RRID:AB_2795846

**Biological samples**		

KB1P(M) mouse mammary tumors	Jaspers et al.^38^	N/A
KB2P mouse mammary tumors	Gogola et al.^9^	N/A

**Chemicals, peptides, and recombinant proteins**		

Olaparib (AZD2281), PARP inhibitor	Syncom, Groningen, the Netherlands	CAS: 763113-22-0
AZD2461, PARP inhibitor	Syncom, Groningen, the Netherlands	CAS: 1174043-16-3

**Critical commercial assays**		

KAPA HTP Library Preparation Kit	Roche	Cat#KK8234
Pierce BCA Protein Assay Kit	Thermo Scientific	Cat#23225
qPCR Lentivirus Titer Kit	Applied Biological Materials	Cat#LV900

**Deposited data**		

WE-seq, LCWG-seq, and RNA-seq of KB1P(M) tumors	European Nucleotide Archive	ENA: PRJEB61242
WE-seq, LCWG-seq, and RNA-seq of KB2P tumors	European Nucleotide Archive	ENA: PRJEB61243
Proteomic/phosphoproteomics data	Proteomics Identification Database	PRIDE: PXD032007
Functional screens sequencing	European Nucleotide Archive	ENA: PRJEB61270
Raw and uncropped data	Mendeley	https://doi.org/10.17632/vkcnb3rrw6.1Mendeley Data:

**Experimental models: Cell lines**		

RPE1-hTERT *BRCA1*^*−/−*^*;TP53*^*−/−*^	Gift from Stephen P. Jackson^57^	N/A
SUM149PT	ATCC	RRID:CVCL_3422
ORG-KB1P4N.1	Duarte et al.^61^	N/A

**Experimental models: Organisms/strains**		

Mouse: FVB/NRj, female	Janvier Labs	N/A
Mouse: (FVB/N X 129/Ola)F1, female	Harlan Olac	N/A

**Oligonucleotides**		

Mouse *Rif1* sgRNA #1 (AGAATTACCTCAGGATTGTC)	This paper	N/A
Mouse *Rif1* sgRNA #2 (CTGGAACACCCGCTAATCA)	This paper	N/A
Mouse *Rif1* sgRNA #1 TIDE PCR Fw (CACTGGGAACCAAACGTAGAT)	This paper	N/A
Mouse *Rif1* sgRNA #1 TIDE PCR Rv (GGAAGCCAGTCCTAACAAATGA)	This paper	N/A
Mouse *Rif1* sgRNA #2 TIDE PCR Fw (CAGCCCTTGCTAGCCTAA)	This paper	N/A
Mouse *Rif1* sgRNA #2 TIDE PCR Rv (CATTCAAGTTTGTGCTCAGGG)	This paper	N/A
Mouse *Shld2* sgRNA #1 (ATCAGTCAGATCCCTGCGTT)	This paper	N/A
Mouse *Shld2* sgRNA #2 (AACCTGAGTGATATGACTAG)	This paper	N/A
Mouse *Shld2* sgRNA #1 TIDE PCR Fw (CCGAAGCACAGAGTGTGAAA)	This paper	N/A
Mouse *Shld2* sgRNA #1 TIDE PCR Rv (CCAGTTTCGCTGCACACTTA)	This paper	N/A
Mouse *Shld2* sgRNA #2 TIDE PCR Fw (TCTGCTCAGGTGGATGAGGA)	This paper	N/A
Mouse *Shld2* sgRNA #2 TIDE PCR Rv (GAAGTTCCGAACGCAGGGAT)	This paper	N/A

**Recombinant DNA**		

Plasmid: lentiGuide-Puro *Rif1* sgRNA #1 (lentiviral)	This paper	N/A
Plasmid: lentiGuide-Puro *Rif1* sgRNA #2 (lentiviral)	This paper	N/A
Plasmid: lentiCRISPR v2-Puro *Shld2* sgRNA #1 (lentiviral)	This paper	N/A
Plasmid: lentiCRISPR v2-Puro *Shld2* sgRNA #2 (lentiviral)	This paper	N/A
Plasmid: pX330-Puro *Shld2* sgRNA #1 (lentiviral)	This paper	N/A
Plasmid: pX330-Puro *Shld2* sgRNA #2 (lentiviral)	This paper	N/A
shRNA library targeting candidate genes (Sigma Mission library TRC) (see Table S4)	This paper	N/A
Plasmid: pLKO.1 - RBMS1 shRNA (mouse, lentiviral)	Sigma Mission Library	TRCN0000096781

**Software and algorithms**		

Cutadapt (version 1.12)	Martin.^74^	https://github.com/marcelm/cutadapt/
BWA (version 0.7.15)	Li.^75^	https://bio-bwa.sourceforge.net/
Picard (version 2.5.0)	Broad GATK	https://broadinstitute.github.io/picard/
Freebayes (version 1.0.2)	Garrison and Marth.^76^	https://github.com/freebayes/freebayes/
SnpEff (version 4.3)	Cingolani et al.^77^	http://pcingola.github.io/SnpEff/
DELLY (version 0.9.1)	Rausch et al.^78^	https://github.com/dellytools/delly
QDNAseq (version 1.26.0)	Scheinin et al.^79^	https://github.com/ccagc/QDNAseq
RUBIC (version 1.0.2)	van Dyk et al.^47^	https://github.com/NKI-CCB/RUBIC
STAR (version 2.5.2b)	Dobin et al.^80^	https://github.com/alexdobin/STAR
featureCounts (version 1.5.0)	Liao et al.^81^	https://subread.sourceforge.net/
edgeR (version 3.32.1)	Robinson et al.^82^	https://bioconductor.org/packages/release/bioc/html/edgeR.html
Limma-voom (version 3.46.0)	Law et al.^83^	https://bioconductor.org/packages/release/bioc/html/limma.html
DIDS (version 0.9.1)	de Ronde et al.^44^	https://github.com/NKI-CCB/dids/
DriverNet (version 1.30.0)	Bashashati et al.^53^	https://www.bioconductor.org/packages/release/bioc/html/DriverNet.html
MaxQuant	Cox et al.^84^	https://www.maxquant.org/
ImageJ software64	Rueden et al.^85^	N/A
ImageJ ColonyArea plugin	Guzmán et al.^86^	N/A
ImageJ macro for the analysis of DNA damage-induced foci	Xu et al.^17^	N/A
MAGeCK	Li et al.^87^	https://github.com/liulab-dfci/MAGeCK
TIDE (Tracking of Indels by Decomposition)	Brinkman et al.^88^	https://tide.nki.nl/

### Resource availability

#### Lead contact

Further information and requests for resources and reagents should be directed to and will be fulfilled by the Lead Contact, Jos Jonkers (j.jonkers@nki.nl).

#### Materials availability

Materials associated with this study are available upon request from the lead contact.

### Experimental model and subject details

#### Cell lines

KB1P (KB1P-G3)^38^ and KB2P (KB2P-3.4)^89^ mouse tumor-derived cell lines were previously established and were grown in DMEM/F12 (Gibco) supplemented with 10% FBS and 50 units/ml penicillin-streptomycin (Gibco), containing 5 μg/mL Insulin (Sigma), 5 ng/mL cholera toxin (Sigma) and 5 ng/mL murine epidermal growth factor (Sigma), under low oxygen conditions (3% O2, 5% CO_2_ at 37°C). RPE1-hTERT *BRCA1*^*−/−*^*;TRP53*^*−/−*^ human cell line has been described before^57^ and was grown in DMEM+GlutaMAX (Gibco) supplemented with 10% FBS and 50 units/ml penicillin-streptomycin (Gibco), under low oxygen conditions (3% O2, 5% CO_2_ at 37°C). SUM149PT (RRID:CVCL_3422) human cell line was grown in RPMI1640 (Gibco) medium supplied with 10% FBS and 50 units/ml penicillin-streptomycin (Gibco), under normal oxygen conditions (21% O2, 5% CO_2_, 37°C).

#### Tumor-derived organoids

ORG-KB1P4N.1 tumor-derived organoids were previously established.^61^ Cultures were embedded in Culturex Reduced Growth Factor Basement Membrane Extract Type 2 (BME, Trevigen; 40 mL BME:growth media 1:1 drop in a single well of 24-well suspension plate) and grown in Advanced DMEM/F12 (GIBCO) supplemented with 1M HEPES (GIBCO), GlutaMAX (GIBCO), 50 units/ml penicillin-streptomycin (GIBCO), B27 (GIBCO), 125 mM N-acetyl-L-cysteine (Sigma) and 50 ng/mL murine epidermal growth factor (Sigma). Organoids were cultured under standard conditions (37°C, 5% CO2).

#### Mice

All animal experiments were approved by the Animal Ethics Committee of the Netherlands Cancer Institute (Amsterdam, the Netherlands) and performed in accordance with the Dutch Act on Animal Experimentation (November 2014). Parental FVB (FVB/NRj) and 129/Ola animals were purchased from Janvier Labs and Harlan Olac, respectively, and crossed at the NKI Animal Facility. KB1P(M) and KB2P tumors were generated in female mice. Tumor implantation experiments were performed in syngeneic, wild-type FVB and F1 (first filial generation) FVB:129/Ola females, at the age of 6 weeks. For intervention studies, 6 weeks old female FVB mice were used. Animals were maintained in the animal department of the NKI, housed in individually ventilated cages (IVC) under specific pathogen-free (SPF) conditions, and received food and water ad libitum.

### Method details

#### Induction of PARPi-resistance in KB1P(M) and KB2P tumors

PARPi-naïve and resistant KB1P(M) and KB2P tumors were previously established.^9^^,^^38^ In brief, spontaneous mammary tumors developed from KB1P(M) and KB2P mice were harvested and orthotopically transplanted into multiple wild-type recipient mice (FVB:129/Ola). All treatments were started when tumors reached a size of approximately 200 mm^3^. AZD2461 (100 mg/kg) to KB1P and KB2P and Olaparib (50 mg/kg) to KB1PM were daily treated for 28 days. When tumors recurred to 100% relative tumor volume, the treatment was repeated for another 28 consecutive days until the tumors acquired resistance. Mice were sacrificed when tumors reached a size of 1,500 mm^3^.

#### *In situ* RAD51-IRIF and 53BP1-IRIF assay

Cryopreserved material of PARPi-naïve or -resistant KB1P, KB1PM and KB2P tumors (KB1P(M): 23 naive, 48 resistant; KB2P: 26 naive, 39 resistant) was thawed and orthotopically engrafted into the right mammary fat pad of 6 week-old wild-type syngeneic female mice (KB1P(M) – FVB; KB2P – FVB:129/Ola(F1)). Tumor volume was monitored starting from two weeks after transplantations and calculated using the following formula: 0.5 x length x width^2^. When tumors reached approximately 500 mm^3^ (100% relative tumor volume), they were locally irradiated using a CT-guided high precision cone beam micro-irradiator (X-RAD 225Cx) or left untreated (control). Two different factors were tested to optimize the assay: IR dosage (15 and 24 Gy) and post-irradiation incubation time (1–6 hr). We did not observe significant differences in RAD51 IRIF formation between the two IR dosages, and the highest accumulation of RAD51 foci was detected 2 hours after induction of DNA damage. Based on these results, we analyzed RAD51 IRIF in KB1P(M) and KB2P tumors 2 hr after irradiation with 15Gy. Post irradiation, tumors were isolated and part of the tissue was immediately fixed in 4% (w/v) solution of formaldehyde in PBS (remaining tissue was fresh frozen for the proteomic and phosphoproteomic analyses). 5 μm-thick FFPE (formalin-fixed paraffin-embedded) tissue sections were then used for immunofluorescence. Following deparaffinization (70°C, 20 min), tissues were rehydrated and cooked in DakoTarget Retrieval Solution pH 9 (#S236784, Dako) for 20 min in a microwave at ∼600W, to allow antigen retrieval. Next, tissue permeabilization was achieved by incubating samples in 0.2% (v/v) Triton X-100 in PBS for 20 min and followed by 1 hr DNAse (1,000 U/ml; #04536282001, Roche) treatment at 37°C. Blocking was done for 30 min in staining buffer (1% (w/v) BSA, 0.15% (w/v) glycine and 0.1% (v/v) Triton X-100 in PBS). Subsequent incubation with primary antibodies was carried out overnight at 4°C, and later with secondary antibodies for 1 hr at room temperature. The following antibodies (diluted in staining buffer) were used in this assay: rabbit polyclonal anti-RAD51 (kind gift from R. Kanaar, Erasmus MC, Rotterdam; 1:5,000), rabbit polyclonal anti-53BP1 (#ab21083, Abcam; 1:1,000), goat polyclonal anti-rabbit, Alexa Fluor® 658-conjugated (#A11011, Thermo Fisher Scientific; diluted 1:1,000). Samples were mounted with VECTASHIELD Hard Set Mounting Media with DAPI (#H-1500; Vector Laboratories). Images were taken with Leica SP5 (Leica Microsystems) confocal system equipped with a x100 objective and image stacks (∼6 slices) were analyzed using an in-house developed ImageJ macro to automatically and objectively quantify IR-induced foci, as described before.^17^^,^^85^ Briefly, nuclei were segmented by thresholding the (median-filtered) DAPI signal, followed by a watershed operation to separate touching nuclei. For each *z*-stack the maximum-intensity projection of the foci signal was background-subtracted using a difference of Gaussians method. Next, for every nucleus, foci candidates were identified as locations where the resulting pixel values exceeded the background by a factor (typically 25x) times the median standard deviation of all nuclei in the image. Additional filters for discriminating for foci size, nucleus size (to eliminate stromal cells) and absolute brightness were applied. Results were validated by visual inspection. Visualization as well as quantification of foci was done in a blinded fashion. For each sample, five random areas (246 × 246 μm; on average 125 cells per area) were imaged and analyzed. A cell was considered positive if contained >5 nuclear foci. KP tumor was used as a positive control in this assay.

#### Immunohistochemistry (IHC)

All IHC stainings were performed on FFPE material. Tissues were formalin-fixed overnight and paraffin-embedded by routine procedures. Tissue sections were boiled for 30 min in citrate buffer pH 6.0 (#CBB 999, Scytek Laboratories) (CD45, B220, PD-1), in Tris (#252859, Sigma) EDTA (Sigma; EDS) (CD3, F4/80), or in HIER Tris EDTA (PD-L1) to facilitate antigen retrieval. Next, staining was carried out by using 3% (v/v) H_2_O_2_ solution in methanol for blocking endogenous peroxidase activity for 20 min and 4% BSA plus 5% normal goat serum (NGS) in PBS as a blocking buffer for 30 min. Primary antibodies were diluted in 1.25% NGS plus 1% BSA in PBS, and applied on the samples overnight, at 4°C (anti-CD45, #ab10558, Abcam 1:200; anti-CD3, #RM-9107, Thermo Fisher Scientific, 1:600; anti-B220, #557390, BD Pharmingen, 1:4000; anti-F4/80, #70076, 1:1000; anti-PD-L1, #64988S, Cell Siganaling, 1:50; anti-PD-1, #18106-1-AP, Proteintech, 1:150). Samples previously incubated with primary rabbit antibodies (CD45, CD3, F4/80, PD-L1, PD-1) were incubated with EnVision+ System- HRP Labeled Polymer Anti-Rabbit (#K4003,Dako) for 30 min. Samples stained with rat antibody (B220) were incubated with Goat-α-Rat-Biotynated secondary antibody (#3052-08, SouthernBiotech, 1:150), diluted in 1.25% NGS/1% BSA in PBS, for 30 min, followed by incubation with streptavidin conjugated to horseradish peroxidase (1:200; 1.25% NGS/1% BSA in PBS; 30 min) (#P0397, Dako). For visualization of all samples, DAB (#D5905, Sigma), H_2_O_2_ (#A-31642, Sigma, 1:1,250) and hematoxylin counterstaining were applied. All slides were digitally processed using the Aperio ScanScope (Aperio, Vista, CA, USA).

#### Whole-exome sequencing (WE-seq)

Genomic DNA was isolated from fresh frozen tumor tissue using a standard Proteinase K and phenol:chloroform extraction and sheared to approximately 300 bp fragments using Covaris S2 sonicator. Next, 500–1000 ng of sheared DNA was used as a template for a 6-cycle PCR to construct a fragmented library using the KAPA HTP Library Preparation Kit (Roche). Exome enrichment was performed using SeqCap EZ Enrichment Kit (Roche) according to the manufacturer’s protocol (SeqCap EZ Library SR User’s Guide, v5.3). Samples were sequenced on an Illumina HiSeq2500 (Illumina). Adapters in the resulting reads (100 base paired-end reads) were trimmed using Cutadapt^74^ (version 1.12) and the trimmed reads were aligned to the GRCm38 reference genome using BWA^75^ (version 0.7.15). The resulting alignments were sorted and marked for duplicates with Picard tools (version 2.5.0). Freebayes variant caller^76^ (version 1.0.2) was used to identify SNVs and Indels for each sample with the mode of pooled-continuous (min-alternate-fraction = 0.1, min-alternate-count = 3, and min-coverage = 10) and resulting variants were annotated by SnpEff (version 4.3).^77^ SNVs and Indels identified in FVB/NJ mice were obtained from Sanger Mouse Genome Project^90^ and used to discard germline variants in our tumor samples. SNVs and Indels that were only identified in resistant tumors and predicted to be high-impact mutations by SnpEff were considered resistant tumor-specific alterations with functional effects and were used for downstream analysis. Structural variants were identified by DELLY^78^ (version 0.9.1) for each pair of resistant and matched naive tumors and resulting variants were annotated by SnpEff. High-impact SVs predicted by SnpEff were used for downstream analysis.

#### Low-coverage whole-genome sequencing (LCWG-seq)

Genomic DNA was isolated from fresh-frozen tumor material using standard phenol:chloroform extraction. LCWG-seq was performed using double-stranded DNA (dsDNA) and quantified with the Qubit® dsDNA HS Assay Kit (Invitrogen, #Q32851). Library preparation was performed with 1 μg of DNA and KAPA HTP Library Preparation Kit (KAPA Biosystems, #KK8234). Samples were sequenced on an Illumina HiSeq2500. Resulting reads (50 base single-end reads for KB1P and KB1PM and 65 base single-end reads for KB2P) were trimmed, sorted and marked for duplicates using the same pipeline as for the WES. The resulting alignments were analyzed to generate segmented profile differences between matched (naive/resistant) samples derived from the same tumor donor using the QDNAseq^79^ (version 1.26.0) and QDNAseq.mm10 (version 1.4.0) R packages (bin size = 50K). To identify regions with recurrent copy number difference (naive vs. resistant), we iteratively ran RUBIC^47^ (version 1.0.2) with default cutoff for calling amplifications and deletions (focal threshold = 1e+08, min probes = 4, FDR < 0.25, amp.level and del.level = 0.1).

#### RNA sequencing (RNA-seq)

Fresh frozen tumor tissue was placed in 1 mL of TRIsure reagent (#BIO-38032, Bioline) and tissue lysis was achieved by high-speed shaking with stainless steel beads for 10 min, 50 Hz at room temperature (TissueLyser LT, Qiagen). Homogenized tissue lysates were further processed according to the TRIsure manufacturer’s protocol. Strand-specific libraries were generated using the TruSeq Stranded mRNA sample preparation kit (Illumina Inc., San Diego, RS-122-2101/2) according to the manufacturer’s instructions. Samples were sequenced on an Illumina HiSeq2000. The resulting reads (50 base single-end reads) were trimmed using Cutadapt^74^ (version 1.12) and aligned to the GRCm38 reference genome using STAR (version 2.5.2b).^80^ To identify differentially expressed (DE) genes, gene expression counts were first generated by featureCounts^81^ (version 1.5.0) using gene definitions from Ensembl GRCm38 (version 76). Genes with counts per million (CPM) larger than one in at least 10% of samples were used for further analysis. Trimmed mean of M-value (TMM) normalization was applied to the data using edgeR^82^ (version 3.32.1) and Limma-voom^83^ (version 3.46.0) was used to correct for the donor effect and identify the differentially expressed genes between naive vs. resistant tumors (FDR < 0.25 for KB1P and FDR<0.05 for KB2P, Log2 fold change>0.5). Because of the intratumoral heterogeneity, we additionally applied DIDS (version 0.9.1) for the detection of subgroup markers in resistant populations (p < 0.05).^44^

#### Selection of previously reported PARPi resistance factors

The list of resistance-associated factors was generated based on previous reports.^7^ We excluded *SLFN11*^13^ which does not have a mouse ortholog and *Shld3* and *Radx* which were not expressed in our mouse cohort. In total, 25 genes were analyzed in our omics datasets (Table S1).

#### Selection of DNA damage response (DDR)-related genes

The DDR gene set was obtained from the previous study that was generated based on merging the gene lists from the previous papers and the NCBI search by terms of “DNA repair”, “DNA damage response”, “DNA replication”, and “telomere-associated genes”.^9^

#### Gene set over-representation analysis

Gene set over-representation analysis was performed using Fisher’s exact test for genes with significant upregulation and downregulation (RNA-seq) and focal gains and losses (CNV-seq) in resistant tumors compared to naive tumors. The significant gene sets were identified as the ones with FDR<0.25.

#### Driver gene analysis

For genes with resistant tumor-specific mutations and copy number variations as mentioned above, DriverNet^53^ (version 1.30.0) was used to infer potential driver genes by assessing the impact of alterations on the expression network. Protein-protein interactome (PPI) to construct an expression network was obtained by orthologue mapping of human PPI merged from multiple PPI databases.^91^^,^^92^^,^^93^^,^^94^^,^^95^ The genes with more than 1.5-fold changes in expression were defined as genes showing outlying expression and used to assess the impact of mutations in the expression network. p-value was computed by gene-based randomization of 1000 times and genes with p-value <0.05 were selected as potential drivers.

#### Mass spectrometry (MS)-based proteomics

For the global proteomic and phosphoproteomic analyses of KB1P(M) tumors, we used previously published proteomics dataset generated by MS (PRIDE accession code: PXD032007).^96^ For phosphoproteomic analysis, For phosphoproteomic analysis, MaxQuant^84^ phosphosite quantification data (Phospho (STY)Sites.txt) was log2-transformed, normalized on the median intensity of all identified phosphosites and replicates averaged favoring data presence. For global protein expression analysis, MaxQuant LFQ Intensity^84^ was log2-transformed and replicates were averaged. limma^48^ was used for differential expression analysis.

#### *Shld2* gene-editing

For CRISPR/Cas9-mediated genome editing of *Shld2,* sgRNAs were cloned into a modified version of the lentiCRISPR v2 backbone (RRID: Addgene_52961) in which a puromycin resistance ORF was cloned under the hPGK promoter, or into the pX330.puro backbone (Addgene #110403). Cloning of sgRNAs into the lentiCRISPR v2 backbone was carried out by melting the custom DNA oligos (Microsynth) at 95°C for 5 min, followed by annealing at RT for 2h and subsequently ligation with T4 ligase (NEB) into the BsmBI*-*digested (Fermantas) backbone. Cloning of sgRNAs into the pX330.puro backbone was performed similarly by ligating the previously annealed oligos into the BbsI-HF-digested backbone with T4 ligase (NEB). KB2P tumor-derived cells were transduced with lentiviral supernatants of the cloned lentiCRISPR v2 constructs and KB1P cells were transfected with the generated pX330.puro plasmids using a transfection reagent (TransIT-LT1 from Mirus) following the manufacturer’s protocol. All constructs' sequences were verified by Sanger sequencing. Gene editing was confirmed by TIDE.^88^

#### *Rif1* gene-editing

For CRISPR/Cas9-mediated genome editing of *Rif1* sgRNAs were cloned into lentiGuide-Puro backbone (RRID: Addgene_52963). Cloning of sgRNAs into the lentGuide-Puro backbone was carried out by melting the custom DNA oligos (IDT) at 95°C for 5 min, followed by annealing at RT for 2h and subsequently ligation with T4 ligase (NEB) into the BsmBI*-*digested (NEB) backbone. KB2P tumor-derived cells expressing Cas9 were transduced with lentiviral supernatants of the cloned lentGuide-Puro *Rif1* sgRNAs as well as with. Gene editing was confirmed by TIDE^88^ and Western blot.

#### Lentiviral transductions

Lentiviral stocks, pseudotyped with the VSV-G envelope, were generated by transient transfection of HEK293FT cells.^87^ Production of integration-deficient lentivirus (IDLV) stocks was carried out in a similar fashion, with the exception that the packaging plasmid contains a point mutation in the integrase gene (psPAX2, gift from Bastian Evers). For transduction of tumor-derived organoids, Lentiviral titers were determined using the qPCR Lentivirus Titration Kit (Applied Biological Materials), following the manufacturer’s instructions. Cells were incubated with lentiviral supernatants overnight in the presence of polybrene (8 μg/mL). Tumor-derived organoids were transduced according to a previously established protocol.^61^ Antibiotic selection was initiated right after transduction for cells, 24h after transduction in organoids, and was carried out for 3 consecutive days.

#### Long-term clonogenic assays

Long-term clonogenic assays were always performed in 6-well plates. KB1P and KB2P tumor-derived cells were seeded at low density to avoid contact inhibition between the clones (4,000 and 3,000 cells/well, respectively). Control untreated plates were fixed with 4% formaldehyde between days 7 and 8 and treated plates between days 8 and 14. For the quantification, cells were stained with 0.1% crystal violet and analyzed in an automated manner using the ImageJ ColonyArea plugin.^85^^,^^86^

#### Functional genetic enrichment screens

We have generated a focused shRNA library targeting the human candidate genes plus non-essential genes as controls, resulting in a total of 1025 genes. We selected 5 hairpins per gene, less when 5 weren’t available, resulting in 5062 lentiviral hairpins (pLKO.1) from the Sigma Mission library (TRC 1.0 and 2.0) (Table S4). This library was then used to generate pools of lentiviral shRNAs which were then transduced in RPE1-hTERT *BRCA1*^*−/−*^*;TRP53*^*−/−*^ and SUM149PT human cells, as described in the section lentiviral transductions. Lentiviral transductions were carried out using a multiplicity of transduction (MOI) of 0.3, in order to ensure that each cell only gets incorporated with one only sgRNA. After transduction, the cells stably expressing integrated shRNA were selected with puromycin. After selection, cells were collected (T0) or seeded in the presence of PARPi (SUM149PT:100.000 cells p/15 cm plate, 10nM olaparib; RPE1-hTERT *BRCA1*^*−/−*^*;TRP53*^*−/−*^: 50.000 cells p/15 cm plate, 50nM olaparib). The total number of cells used in a single screen was calculated as follows: library complexity x coverage (1000x). Triplicates were carried out for both cell lines. Cells were kept in culture for 3 weeks and the medium was refreshed every 5 days. In the end of the screen, or at T0, cells were pooled and genomic DNA was extracted (QIAmp DNA Mini Kit, Qiagen). shRNA sequences were retrieved by a two-step PCR amplification, as described before.^17^^,^^97^ To maintain screening coverage, the amount of genomic DNA used as an input for the first PCR reaction was taken into account (6 μg of genomic DNA per 10^6^ genomes, 1 μg/PCR reaction). The resulting PCR products were purified using MiniElute PCR Purification Kit (Qiagen) and submitted for Illumina sequencing. Sequence alignment and dropout analysis was carried out using the algorithm MaGECK.^98^

#### *In vivo* intervention studies

Tumor organoids transduced with shRNA^61^^,^^97^ targeting RBMS1 were collected, incubated with TripLE at 37°C for 10 min, dissociated into single cells, resuspended in tumor organoid medium, filtered with 70μm nilon filters (Corning) and mixed in a in complete mouse media/BME mixture (1:1). ORG-KB1P4N.1 organoid suspensions contained a total of 40.000 cells per 40 μl of media/BME mixture, and were injected in the fourth right mammary fat pad of wild-type FVB/N mice. Mammary tumor size was determined by caliper measurements (length and width in millimeters), and tumor volume (in mm^3^) was calculated by using the following formula: 0.5 × length × width^2^. Upon tumor outgrowth to approximately 75 mm^3^ mice were treated with vehicle or 100 mg/kg olaparib intraperitoneally for 28 consecutive days. Animals were sacrificed with CO2 when the tumor volume reached 1,500 mm^3^.

### Quantification and statistical analysis

High-throughput genomics, transcriptomics, proteomics and phosphoproteomics data were analyzed as described in relevant method details sections. Statistical analysis of long-term clonogenic assays was performed using 2-way ANOVA followed by Dunnett’s test. For the analysis of RAD51/53BP1 IRIF data we used the two-tailed Mann-Whitney *U* test. ^∗∗∗∗^ p < 0.0001, ^∗∗∗^ p < 0.001, ^∗∗^ p < 0.01.

## Data Availability

• Raw sequencing data of WE-seq, LCWG-seq and RNA-seq reported in this paper are available in European Nucleotide Archive (ENA) under accession number ENA: PRJEB61242 for KB1P(M) tumors and ENA: PRJEB61243 for KB2P tumors. Raw sequencing data of functional screens is available under accession number ENA: PRJEB61270. Global and phosphoproteomic data are available in the Proteomics Identification Database (PRIDE) under accession number PRIDE: PXD032007. • This paper does not report original code. • Any additional information required to reanalyze the data reported in this paper is available from the lead contact upon request.

## Supplemental information

Document S1. Figures S1–S4 and Table S1

Table S2. Genes with genomic and transcriptional alterations in KB1P RAD51-positive, KB1P RAD51-negative, and KB2P PARPi-resistant tumors, related to Figure 4

Table S3. Hallmark and KEGG gene sets represented by genes with genomic and transcriptional alterations in KB1P RAD51-positive, KB1P RAD51-negative, and KB2P PARPi-resistant tumors, related to Figure 4

Table S4. List of genes used for *in vitro* loss-of-function screens and shRNA sequences designed to target each gene, related to Figure 4

Document S2. Article plus supplemental information
