# Supplementary material for: Multi-omics analysis reveals distinct non-reversion mechanisms of PARPi resistance in BRCA1- versus BRCA2-deficient mammary tumors
Source: Cell Rep. 2023 May 19;42(5):112538. doi: 10.1016/j.celrep.2023.112538 (PMC10242444; doi:10.1016/j.celrep.2023.112538)
Supplement: Document S1. Figures S1–S4 and Table S1 [file mmc1.pdf]

Cell Reports, Volume *42*

Supplemental information

Multi-omics analysis reveals distinct non-reversion mechanisms of PARPi resistance in BRCA1- versus BRCA2-deficient mammary tumors

Jinhyuk Bhin, Mariana Paes Dias, Ewa Gogola, Frank Rolfs, Sander R. Piersma, Roebi de Bruijn, Julian R. de Ruiter, Bram van den Broek, Alexandra A. Duarte, Wendy Sol, Ingrid van der Heijden, Christina Andronikou, Taina S. Kaiponen, Lara Bakker, Cor Lieftink, Ben Morris, Roderick L. Beijersbergen, Marieke van de Ven, Connie R. Jimenez, Lodewyk F.A. Wessels, Sven Rottenberg, and Jos Jonkers

Supplementary information

Figure S1

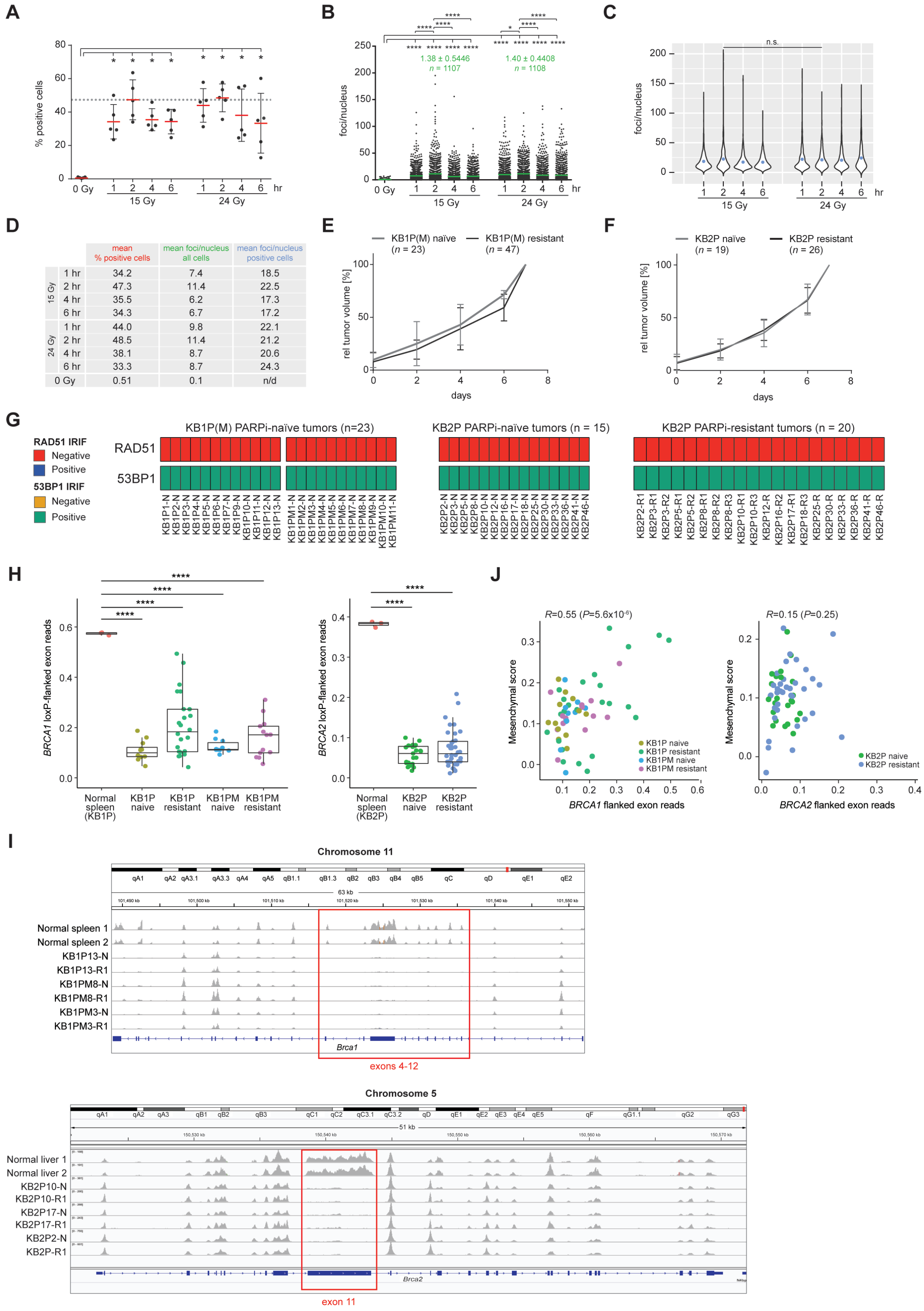

		mean % positive cells	mean foci/nucleus all cells	mean foci/nucleus positive cells
15 Gy	1 hr	34.2	7.4	18.5
	2 hr	47.3	11.4	22.5
	4 hr	35.5	6.2	17.3
	6 hr	34.3	6.7	17.2
24 Gy	1 hr	44.0	9.8	22.1
	2 hr	48.5	11.4	21.2
	4 hr	38.1	8.7	20.6
	6 hr	33.3	8.7	24.3
0 Gy		0.51	0.1	n/d

Figure S1. Optimization of the RAD51 IRIF formation assay. Related to Figure 1

(A) Percentage of RAD51 positive cells (>5 foci/nucleus) per tumor area (single data point) in a KP tumor irradiated with 0, 15 or 24 Gy, 1-6 hr post-irradiation; *n* = 5 per imaged area; *p<0.05 (two-tailed Mann-Whitney U test, followed by Benjamini-Hochberg (BH) correction); data shown as mean (red line) ± SD of a replicate, the experiment was repeated twice; grey dotted line indicates the mean value of a sample irradiated with 15 Gy and incubated for 2 hr. **(B)** Number of foci per nucleus (single data point) quantified for the total tumor cell population of a KP described in (A); mean values are represented by green lines; ****p<0.0001, statistical analysis as in (A). **(C)** Number of foci per nucleus quantified for positive cell population (>5 foci/nucleus) of a KP tumor described in (A), and represented as violin plots showing the density (width = frequency) of the data; mean values are represented by blue dots; statistical analysis as in (A). **(D)** Summary of analyses represented in (A-C). **(E-F)** Growth curves of KB1P(M) (E) and KB2P (F) tumors, samples were irradiated at day 7. **(G)** Outcome of the RAD51 and 53BP1 IRIF assays for PARPi-naïve KB1P(M) and KB2P tumors and for PARPi-resistant KB2P tumors; N – naïve; R – resistant. **(H)** Ratio of the reads mapping to the Brca1/2 exons flanked by loxP recombination sites (exons 4-12 for Brca1; previously referred to as exon 5-13 [S1] and exon 11 for Brca2) among the reads mapping to the gene body of Brca1/2 for all KB1P (*n* = 12 for naïve and *n* = 29 for resistant tumors), KB1PM (*n* = 11 for naïve and n = 15 for resistant tumors), and KB2P tumor (*n* = 25 for naïve and n = 37 for resistant tumors) samples as well as normal tissues (spleen and liver controls). ****p<0.0001 (two-tailed Mann-Whitney U test, followed by BH correction). Data are represented as mean ± s.d. **(I)** Representative images of exome sequencing reads for the Brca1/2 genes in KB1P(M) and KB2P tumor samples and normal tissues (spleen 1/2 and liver 1/2; controls), showing that deletions of flanked exons (marked in red; Brca1 – exons 4-12, Brca2 – exon 11) are preserved in PARPi-naïve and PARPi-resistant tumor samples. **(J)** Correlation between ratios of reads in the deleted exons flanked by loxP as mentioned in (H) and mesenchymal scores computed by singscore [S2] using the previously reported mesenchymal signature gene sets [S3].

Figure S2

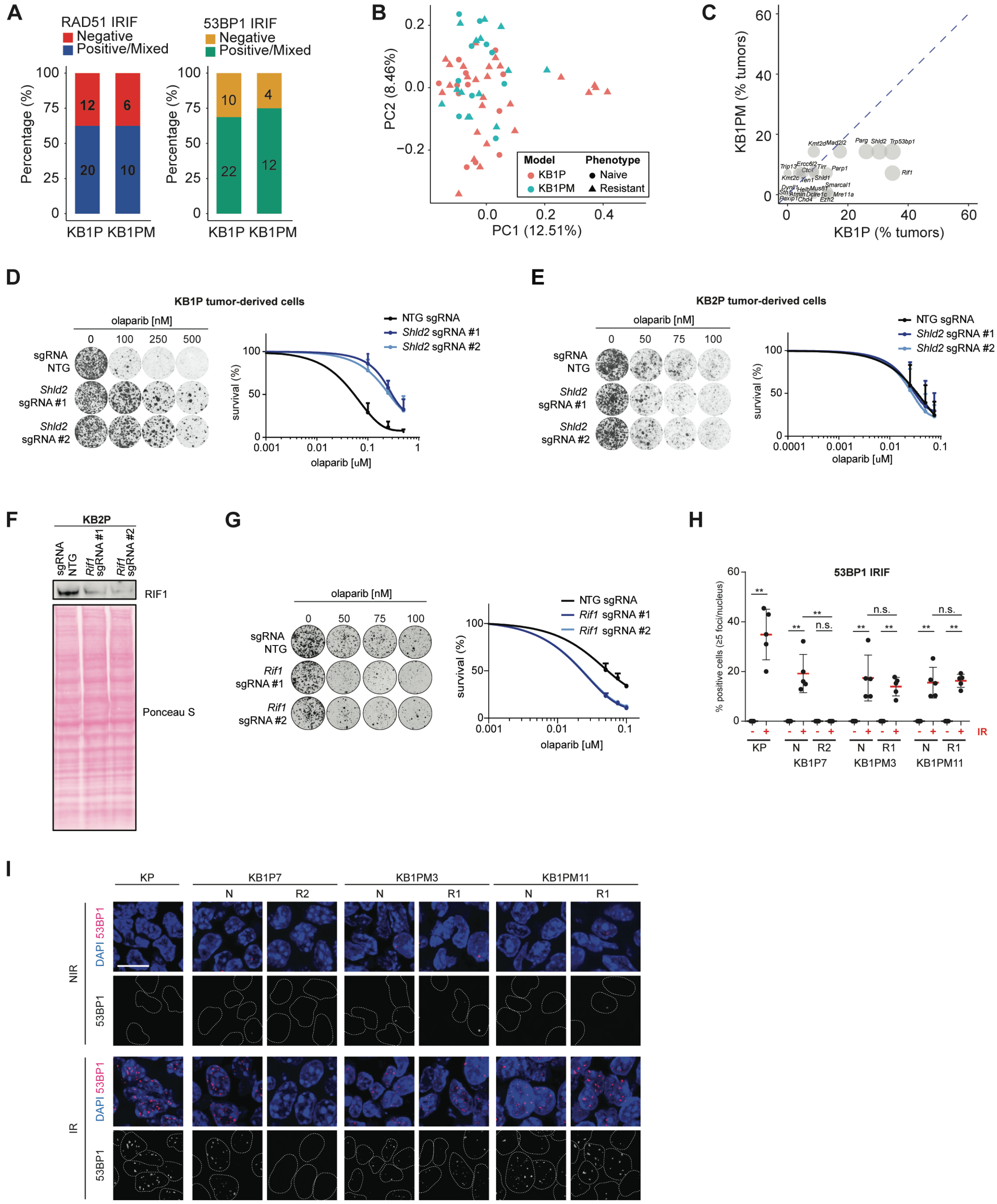

A
RAD51 IRIF
Negative
Positive/Mixed
53BP1 IRIF
Negative
Positive/Mixed
Percentage (%)
KB1P KB1PM
B
PC2 (8.46%)
PC1 (12.51%)
Model
KB1P
KB1PM
Phenotype
Naive
Resistant
C
KB1PM (% tumors)
KB1P (% tumors)
D
KB1P tumor-derived cells
olaparib [nM]
sgRNA NTG
Shld2 sgRNA #1
Shld2 sgRNA #2
NTG sgRNA
survival (%)
olaparib [uM]
E
KB2P tumor-derived cells
F
KB2P
RIF1
Ponceau S
G
Rif1 sgRNA #1
Rif1 sgRNA #2
H
53BP1 IRIF
% positive cells (≥5 foci/nucleus)
IR
KP
KB1P7
KB1PM3
KB1PM11
n.s.
I
NIR
DAPI 53BP1
53BP1

Figure S2. Trp53bp1 gene expression correlates with 53BP1 IRIF. Related to Figure 2

(A) Percentage of RAD51 IRIF-positive and -negative tumors in PARPi-resistant KB1P and KB1PM tumors. **(B)** Principal component analysis of gene expression data ($\log_2$[count per million;CPM]) for KB1P (pink) and KB1PM (cyan) tumors. **(C)** Scatter plot comparing the alteration frequency of each PARPi resistance factor in KB1P and KB1PM tumors. The size of the circle is proportional to the sum of the alteration frequency of the two resistant tumor types compared and circles are colored if statistically significant (Fisher's exact test, $P<0.05$). **(D-E)** Representative images (left) and quantification (right) of clonogenic assays in the presence of olaparib in KB1P (D) and KB2P (E) tumor-derived cells modified by CRISPR-Cas9 with sgRNAs targeting Shld2 or non-targeting (NTG) controls. **(F)** Western blot analysis of RIF1 in whole-cell lysates. Ponceau S staining was used as loading control. **(G)** Representative images (left) and quantification (right) of clonogenic assays in the presence of olaparib in KB2P tumor-derived cells modified by CRISPR-Cas9 with sgRNAs targeting Rif1 or nontargeting (NTG) controls. **(H-I)** Quantification (H) and representative images (I) of 53BP1 IRIFs for the different matched KB1P(M) tumor pairs; IR – irradiated, NIR – non irradiated; scale bar, 100 µm; data in (H) represented as the percentage of positive cells (≥5 foci/nucleus) per imaged area (single data point, typically 100-200 cells/area); *n* = 5 per imaged area. Data are represented as mean ± s.d.; $^{**}p<0.01$, n.s. not significant (two-tailed Mann-Whitney U test, followed by BH correction).

Figure S3

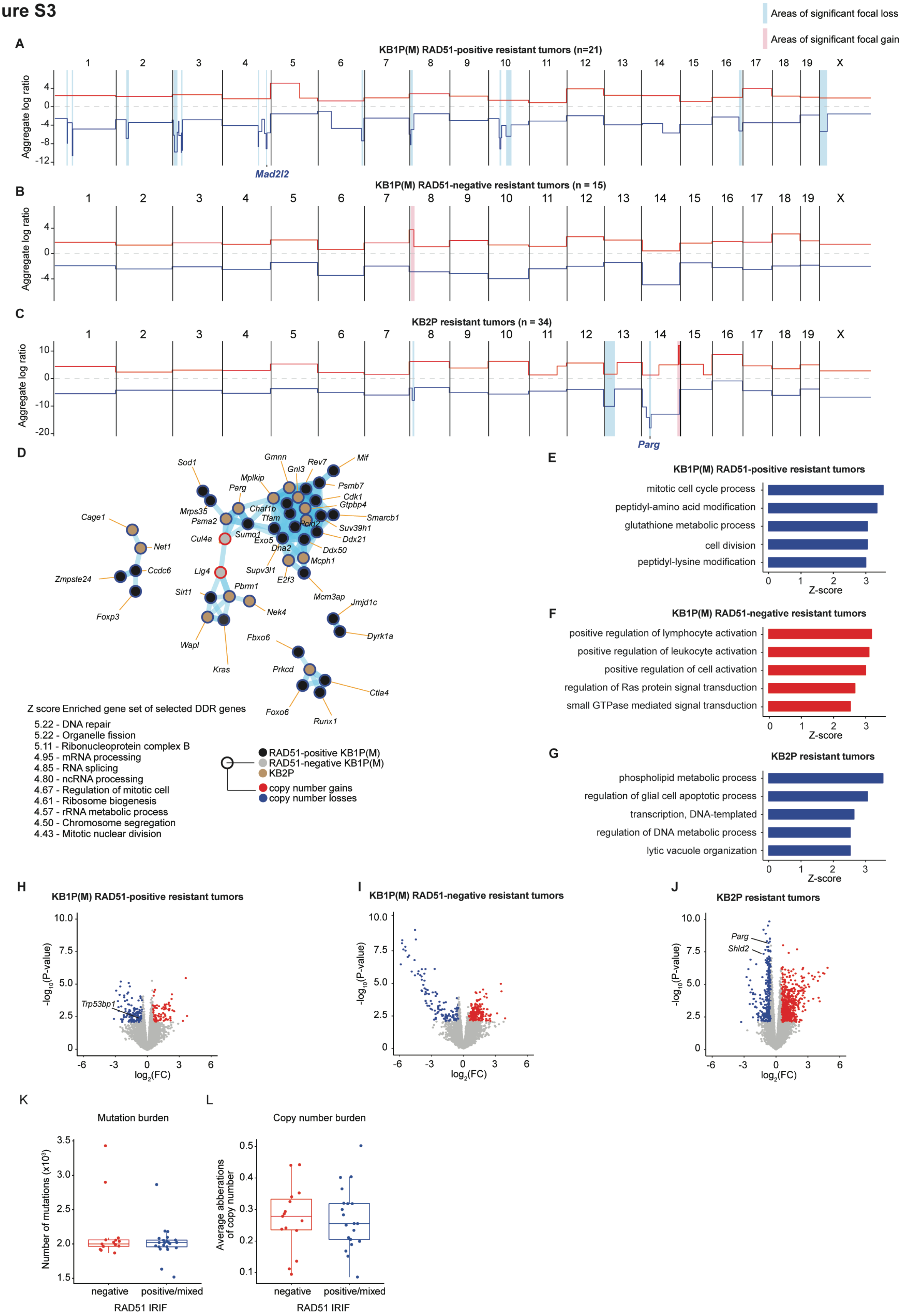

Areas of significant focal loss
Areas of significant focal gain
A
KB1P(M) RAD51-positive resistant tumors (n=21)
Aggregate log ratio
Mad2l2
B
KB1P(M) RAD51-negative resistant tumors (n = 15)
Aggregate log ratio
C
KB2P resistant tumors (n = 34)
Aggregate log ratio
Parg
D
Sod1
Gmnn
Rev7
Mif
Gnl3
Mplkip
Psmb7
Parg
Cdk1
Gtpbp4
Mrps35
Chaf1b
Psma2
Tfam
Smarcb1
Cage1
Pold2
Cul4a
Sumo1
Suv39h1
Exo5
Ddx21
Net1
Dna2
Ddx50
Ccdc6
Lig4
Mcph1
Supv3l1
Zmpste24
E2f3
Pbrm1
Sirt1
Mcm3ap
Jmjd1c
Foxp3
Nek4
Wapl
Dyrk1a
Fbxo6
Kras
Prkcd
Ctla4
Foxo6
Runx1
Z score Enriched gene set of selected DDR genes
5.22 - DNA repair
5.22 - Organelle fission
5.11 - Ribonucleoprotein complex B
4.95 - mRNA processing
4.85 - RNA splicing
4.80 - ncRNA processing
4.67 - Regulation of mitotic cell
4.61 - Ribosome biogenesis
4.57 - rRNA metabolic process
4.50 - Chromosome segregation
4.43 - Mitotic nuclear division
RAD51-positive KB1P(M)
RAD51-negative KB1P(M)
KB2P
copy number gains
copy number losses
E
KB1P(M) RAD51-positive resistant tumors
mitotic cell cycle process
peptidyl-amino acid modification
glutathione metabolic process
cell division
peptidyl-lysine modification
Z-score
F
KB1P(M) RAD51-negative resistant tumors
positive regulation of lymphocyte activation
positive regulation of leukocyte activation
positive regulation of cell activation
regulation of Ras protein signal transduction
small GTPase mediated signal transduction
Z-score
G
KB2P resistant tumors
phospholipid metabolic process
regulation of glial cell apoptotic process
transcription, DNA-templated
regulation of DNA metabolic process
lytic vacuole organization
Z-score
H
KB1P(M) RAD51-positive resistant tumors
-log10(P-value)
Trp53bp1
log2(FC)
I
KB1P(M) RAD51-negative resistant tumors
-log10(P-value)
log2(FC)
J
KB2P resistant tumors
Parg
Shld2
-log10(P-value)
log2(FC)
K
Mutation burden
Number of mutations (x10³)
negative
positive/mixed
RAD51 IRIF
L
Copy number burden
Average abberations of copy number
negative
positive/mixed
RAD51 IRIF

Figure S3. Systematic analysis of genomic and transcriptomics data for PARP-naïve vs PARPi-resistant KB1P(M) and KB2P tumors. Related to Figure 3

(A-C) Identification of focal gains and losses in RAD51-positive (A), RAD51-negative (B) KB1P(M) resistant tumors, and KB2P resistant tumors (C) compared to matched naïve tumors using RUBIC. Genes were highlighted if they are previously reported PARPi-resistance factors. **(D)** Co-functionality network of DDR genes with significant focal gain or loss in RAD51-positive KB1P(M) (black), RAD51-negative KB1P(M) (gray) and KB2P (brown) resistant tumors. Gain (red) or loss (blue) are represented by border color DDR, DNA damage response. Top 10 pathways enriched by the genes in the network are listed with Z-transformed P value. The network construction and pathway analysis were performed by the GenetICA-Network. **(E-G)** Gene set analysis represented by the genes with significant focal gain or loss in PARPi-resistant RAD51-positive KB1P(M) (E), RAD51-negative KB1P(M) (F) and KB2P (G) tumors, detected by RUBIC. Fisher's exact test was performed using gene sets obtained from Gene ontology biological processes. Because only focal losses were detected in RAD51-positive KB1P(M) resistant tumors, gene set analysis was performed for the genes with focal losses (blue). For RAD51-negative KB1P(M) resistant tumors, only one focal gain was detected, gene set analysis was therefore performed only for the genes with focal gain (red). KB2P tumors showed three focal losses and one focal gain. The focal gain was only encoded mostly for non-protein-coding genes with the exception of three genes and thus gene set analysis was performed only for the genes encoded by the areas of focal losses. The top 5 gene sets based on the Z-transformed P-value are shown. **(H-J)** Volcano plots of differentially expressed genes (DEGs) in PARPi-naïve vs PARPi-resistant RAD51-positive KB1P(M) (H), RAD51-negative KB1P(M) (I) and KB2P (J) tumors. Genes were highlighted if they are previously reported PARPi-resistance factors. **(K)** The number of resistant tumor-specific mutations in RAD51-negative (n = 15) and RAD51-positive/mixed (n = 22) KB1P(M) resistant tumors. **(L)** Average copy number variations in resistant tumors compared to matched naïve tumors in RAD51-negative and RAD51-positive/mixed KB1P(M) resistant tumors. Data are represented as mean ± s.d.

Figure S4

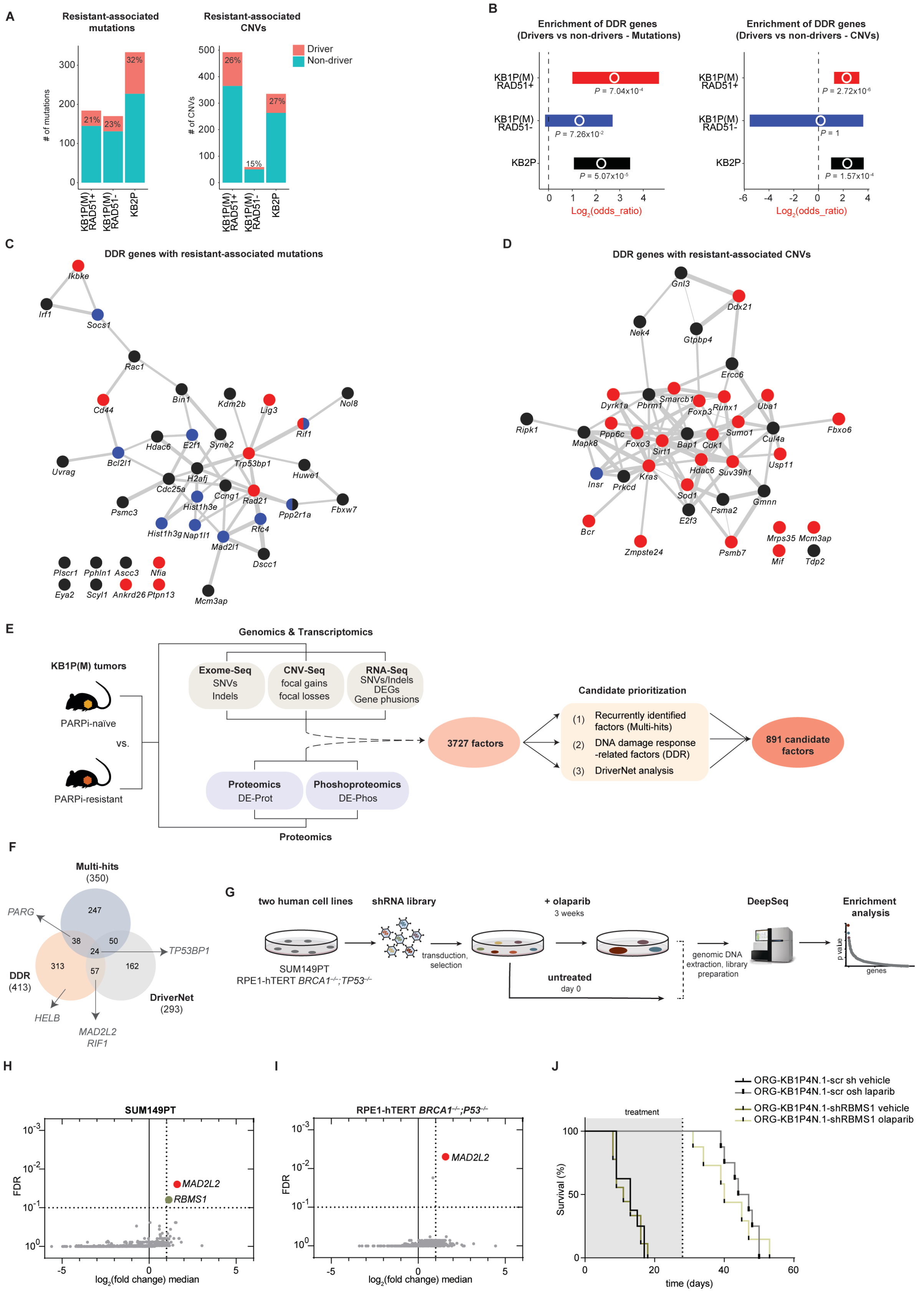
A
Resistant-associated mutations
Resistant-associated CNVs
# of mutations
# of CNVs
21%
23%
32%
26%
15%
27%
Driver
Non-driver
KB1P(M) RAD51+
KB1P(M) RAD51-
KB2P
B
Enrichment of DDR genes (Drivers vs non-drivers - Mutations)
Enrichment of DDR genes (Drivers vs non-drivers - CNVs)
P = 7.04x10-4
P = 7.26x10-2
P = 5.07x10-5
P = 2.72x10-6
P = 1
P = 1.57x10-4
Log2(odds_ratio)
C
DDR genes with resistant-associated mutations
Ikbke
Irf1
Socs1
Rac1
Cd44
Bin1
Kdm2b
Lig3
Nol8
Rif1
Hdac6
E2f1
Syne2
Uvrag
Bcl2l1
Trp53bp1
H2afj
Huwe1
Cdc25a
Ccng1
Rad21
Psmc3
Hist1h3e
Ppp2r1a
Fbxw7
Rfc4
Hist1h3g
Nap1l1
Mad2l1
Dscc1
Plscr1
Pphln1
Ascc3
Nfia
Eya2
Scyl1
Ankrd26
Ptpn13
Mcm3ap
D
DDR genes with resistant-associated CNVs
Gnl3
Ddx21
Nek4
Gtpbp4
Ercc6
Dyrk1a
Pbrm1
Smarcb1
Foxp3
Runx1
Uba1
Ripk1
Fbxo6
Mapk8
Ppp6c
Foxo3
Bap1
Cdk1
Sumo1
Cul4a
Sirt1
Usp11
Hdac6
Suv39h1
Insr
Prkcd
Kras
Sod1
Psma2
Gmnn
E2f3
Bcr
Mrps35
Mcm3ap
Zmpste24
Psmb7
Mif
Tdp2
E
KB1P(M) tumors
PARPi-naïve
vs.
PARPi-resistant
Genomics & Transcriptomics
Exome-Seq
SNVs
Indels
CNV-Seq
focal gains
focal losses
RNA-Seq
SNVs/Indels
DEGs
Gene phusions
Proteomics
DE-Prot
Phoshoproteomics
DE-Phos
Proteomics
3727 factors
Candidate prioritization
(1) Recurrently identified factors (Multi-hits)
(2) DNA damage response -related factors (DDR)
(3) DriverNet analysis
891 candidate factors
F
Multi-hits
(350)
DDR
(413)
DriverNet
(293)
247
38
50
24
313
57
162
PARG
TP53BP1
HELB
MAD2L2
RIF1
G
two human cell lines
SUM149PT
RPE1-hTERT BRCA1-/-;TP53-/-
shRNA library
transduction, selection
+ olaparib
3 weeks
untreated
day 0
genomic DNA extraction, library preparation
DeepSeq
Enrichment analysis
p value
genes
H
SUM149PT
MAD2L2
RBMS1
FDR
log2(fold change) median
I
RPE1-hTERT BRCA1-/-;P53-/-
MAD2L2
J
ORG-KB1P4N.1-scr sh vehicle
ORG-KB1P4N.1-scr osh laparib
ORG-KB1P4N.1-shRBMS1 vehicle
ORG-KB1P4N.1-shRBMS1 olaparib
treatment
Survival (%)
time (days)

Figure S4. Loss-of-function genetic screens are insufficient to validate the different dimensions of our findings. Related to Figure 4

(A) Bar plot representing the number of driver and non-driver genes with high impact on expression of neighbors in a protein-protein interaction network. Driver potential was assessed for the genes with resistance-specific mutations (left) and copy number variations (right) by DriverNet. **(B)** Enrichment of DDR genes in the identified drivers from each resistant tumor group. Log2 odd ratios (proportion of DDR genes in driver genes vs non-driver genes) were presented with 95% confidence intervals. P-values were computed by fisher's exact test. **(C-D)** DDR genes identified as drivers from resistance-associated mutations (C) and CNVs (D) were presented in the co-functionality networks. Node colors indicate genes from different resistant tumor groups (red: KB1P(M) HR-positive resistant tumors, blue: KB1P(M) HR-negative resistant tumors, black: KB2P resistant tumors), **(E)** Schematic diagram showing the process of prioritizing candidate genes for functional screening. To enrich for plausible resistance driver genes, candidates were selected if (1) identified independently by at least two analyses ('multi-hits'), or (2) implicated in the DDR, or (3) identified as high-impact drivers in the protein-protein network using DriverNet. The latter identifies genes which impact the expression of interacting partners or factors that share the same biological pathway and was used to correct for the fact that frequency analyses integrating data across different omic platforms ('multihits') fails to identify events restricted to one biochemical domain (e.g., mutation or phosphorylation), but nonetheless important for driving resistance. WE-seq, LCWG-seq and RNA-seq analysis was included for all tumor collections, including tumors whose RAD51-IRIF status was not determined in KB1P(M) (68 for resistant and 43 for naïve tumors), and proteomics and phosphoproteomics data was generated for some of the KB1P(M) tumors (12 for resistant and 12 for naïve tumors). **(F)** Venn diagram representing the number of genes identified in candidate prioritization analysis. Genes were highlighted if they are previously reported PARPi-resistance factors. **(G)** Outline of functional genetic enrichment screen. Screen was performed in SUM149PT and RPE1-hTERT BRCA1-/-;TP53-/- cells. Surviving cells were collected after 3 weeks and analysis and hit selection was performed using the MaGECK algorithm [S4]. **(H-I)** Plot of distribution log2ratio (fold change (treated versus untreated)) median for all genes versus false discovery rate (FDR) for the screen carried out in SUM149PT (H) and in RPE1-hTERT BRCA1-/-;TP53-/- (I) cells. **(J)** Kaplan–Meier survival curves of mice transplanted with ORG-KB1P4N.1 tumoroids lines modified with indicated shRNA and treated with 100 mg/kg olaparib. End of treatment (28 days) is indicated by a dotted line.

Table S1. Previously reported PARPi resistance factors. Related to Figure 2

Resistance mechanism	Gene	Publications (References)
Loss of trapping/rescue of PARP signaling	PARP1	Pettitt et al., 2013 [S5], Pettitt et al., 2018 [S6]
	PARG	Gogola et al., 2018 [S7]
HR restoration	53BP1	Bouwman et al., 2010 [S8], Bunting et al., 2010 [S9]
	MAD2L2	Boersma et al., 2015 [S10], Xu et al., 2015 [S11]
	RIF1	Escribano-Díaz et al., 2013 [S12], Chapman et al., 2013 [S13], Zimmermann et al., 2013 [S14]
	SHLD1/2	Dev et al., 2018 [S15], Ghezraoui et al., 2018 [S16], Noordermeer et al., 2018 [S17], Gupta et al., 2018 [S18], Findlay et al., 2018 [S19], Gao et al., 2018 [S20]
	CTC1/ STN1/ TEN1	Barazas et al., 2019 [S21], Mirman et al., 2018 [S22]
	HELB	Tkáč et al., 2016 [S23]
	DYNLL1/ ATMIN	Becker et al., 2018 [S24], He et al., 2018 [S25]
	ERCC6L2	Olivieri et al., 2020 [S26], Francica et al., 2020 [S27]
	DCLRE1C	Wang et al., 2014 [S28]
	TIRR	Drané et al., 2017 [S29]
	TRIP13	Clairmont et al., 2020 [S30]
Restoration of fork stability	MRE11	Ray Chaudhuri et al., 2016 [S31], Ying et al., 2012 [S32], Lemaçon et al., 2017 [S33], Schlacher et al., 2011 [S34], Schlacher et al., 2012 [S35]
	EZH2	Rondinelli et al., 2017 [S36]
	MUS81	Rondinelli et al., 2017 [S36]
	CHD4/PAXIP1	Ray Chaudhuri et al., 2016 [S31], Ying et al., 2012 [S32]
	KMT2C/2D	Ray Chaudhuri et al., 2016 [S31], Ying et al., 2012 [S32]
	SMARCAL1	Taglialatela et al., 2017 [S37]

References

1. Liu, X., Holstege, H., Gulden, H., Treur-Mulder, M., Zevenhoven, J., Velds, A., Kerkhoven, R.M., Vliet, M.H., Wessels, L.F.A., Peterse, J.L., et al. (2007). Somatic loss of BRCA1 and p53 in mice induces mammary tumors with features of human BRCA1-mutated basal-like breast cancer. Proceedings of the National Academy of Sciences 104, 12111–12116. 10.1073/pnas.0702969104.

2. Foroutan, M., Bhuva, D.D., Lyu, R., Horan, K., Cursons, J., and Davis, M.J. (2018). Single sample scoring of molecular phenotypes. BMC Bioinformatics 19, 404. 10.1186/s12859-018-2435-4.

3. Mak, M.P., Tong, P., Diao, L., Cardnell, R.J., Gibbons, D.L., William, W.N., Skoulidis, F., Parra, E.R., Rodriguez-Canales, J., Wistuba, I.I., et al. (2016). A Patient-Derived, Pan-Cancer EMT Signature Identifies Global Molecular Alterations and Immune Target Enrichment Following Epithelial-to-Mesenchymal Transition. Clin Cancer Res 22, 609–620. 10.1158/1078-0432.CCR-15-0876.

4. Li, W., Xu, H., Xiao, T., Cong, L., Love, M.I., Zhang, F., Irizarry, R.A., Liu, J.S., Brown, M., and Liu, X.S. (2014). MAGeCK enables robust identification of essential genes from genome-scale CRISPR/Cas9 knockout screens. Genome Biol 15, 554. 10.1186/s13059-014-0554-4.

5. Pettitt, S.J., Rehman, F.L., Bajrami, I., Brough, R., Wallberg, F., Kozarewa, I., Fenwick, K., Assiotis, I., Chen, L., Campbell, J., et al. (2013). A genetic screen using the PiggyBac transposon in haploid cells identifies Parp1 as a mediator of olaparib toxicity. PloS one 8, e61520. 10.1371/journal.pone.0061520.

6. Pettitt, S.J., Krastev, D.B., Brandsma, I., Dréan, A., Song, F., Aleksandrov, R., Harrell, M.I., Menon, M., Brough, R., Campbell, J., et al. (2018). Genome-wide and high-density CRISPR-Cas9 screens identify point mutations in PARP1 causing PARP inhibitor resistance. Nature Communications 9, 1849. 10.1038/s41467-018-03917-2.

7. Gogola, E., Duarte, A.A., de Ruiter, J.R., Wiegant, W.W., Schmid, J.A., de Bruijn, R., James, D.I., Guerrero Llobet, S., Vis, D.J., Annunziato, S., et al. (2018). Selective Loss of PARG Restores PARylation and Counteracts PARP Inhibitor-Mediated Synthetic Lethality. Cancer Cell 33, 1078-1093.e12. 10.1016/j.ccell.2018.05.008.

8. Bouwman, P., Aly, A., Escandell, J.M., Pieterse, M., Bartkova, J., van der Gulden, H., Hiddingh, S., Thanasoula, M., Kulkarni, A., Yang, Q., et al. (2010). 53BP1 loss rescues BRCA1 deficiency and is associated with triple-negative and BRCA-mutated breast cancers. Nature Structural & Molecular Biology 17, 688–695. 10.1038/nsmb.1831.

9. Bunting, S.F., Callén, E., Wong, N., Chen, H.-T., Polato, F., Gunn, A., Bothmer, A., Feldhahn, N., Fernandez-Capetillo, O., Cao, L., et al. (2010). 53BP1 Inhibits Homologous Recombination in Brca1-Deficient Cells by Blocking Resection of DNA Breaks. Cell 141, 243–254. 10.1016/j.cell.2010.03.012.

10. Boersma, V., Moatti, N., Segura-Bayona, S., Peuscher, M.H., van der Torre, J., Wevers, B. a, Orthwein, A., Durocher, D., and Jacobs, J.J.L. (2015). MAD2L2 controls DNA repair at telomeres and DNA breaks by inhibiting 5' end resection. Nature 521, 537–540. 10.1038/nature14216.

11. Xu, G., Ross Chapman, J., Brandsma, I., Yuan, J., Mistrik, M., Bouwman, P., Bartkova, J., Gogola, E., Warmerdam, D., Barazas, M., et al. (2015). REV7 counteracts DNA double-strand break resection and affects PARP inhibition. Nature 521, 541–544. 10.1038/nature14328.

12. Escribano-Díaz, C., Orthwein, A., Fradet-Turcotte, A., Xing, M., Young, J.T.F., Tkáč, J., Cook, M.A., Rosebrock, A.P., Munro, M., Canny, M.D., et al. (2013). A Cell Cycle-Dependent Regulatory Circuit Composed of 53BP1-RIF1 and BRCA1-CtIP Controls DNA Repair Pathway Choice. Molecular Cell 49, 872–883. 10.1016/j.molcel.2013.01.001.

13. Chapman, J.R., Barral, P., Vannier, J.B., Borel, V., Steger, M., Tomas-Loba, A., Sartori, A.A., Adams, I.R., Batista, F.D., and Boulton, S.J. (2013). RIF1 Is Essential for 53BP1-Dependent Nonhomologous End Joining and Suppression of DNA Double-Strand Break Resection. Molecular Cell 49, 858–871. 10.1016/j.molcel.2013.01.002.

14. Zimmermann, M., Lottersberger, F., Buonomo, S.B., Sfeir, A., and de Lange, T. (2013). 53BP1 regulates DSB repair using Rif1 to control 5’ end resection. Science (New York, N.Y.) 339, 700–704. 10.1126/science.1231573.

15. Dev, H., Chiang, T.-W.W., Lescale, C., de Krijger, I., Martin, A.G., Pilger, D., Coates, J., Sczaniecka-Clift, M., Wei, W., Ostermaier, M., et al. (2018). Shieldin complex promotes DNA end-joining and counters homologous recombination in BRCA1-null cells. Nature Cell Biology 20, 954–965. 10.1038/s41556-018-0140-1.

16. Ghezraoui, H., Oliveira, C., Becker, J.R., Bilham, K., Moralli, D., Anzilotti, C., Fischer, R., Deobagkar-Lele, M., Sanchiz-Calvo, M., Fueyo-Marcos, E., et al. (2018). 53BP1 cooperation with the REV7–shieldin complex underpins DNA structure-specific NHEJ. Nature 560, 122–127. 10.1038/s41586-018-0362-1.

17. Noordermeer, S.M., Adam, S., Setiaputra, D., Barazas, M., Pettitt, S.J., Ling, A.K., Olivieri, M., Álvarez-Quilón, A., Moatti, N., Zimmermann, M., et al. (2018). The shieldin complex mediates 53BP1-dependent DNA repair. Nature 560, 117–121. 10.1038/s41586-018-0340-7.

18. Gupta, R., Somyajit, K., Narita, T., Maskey, E., Stanlie, A., Kremer, M., Typas, D., Lammers, M., Mailand, N., Nussenzweig, A., et al. (2018). DNA Repair Network Analysis Reveals Shieldin as a Key Regulator of NHEJ and PARP Inhibitor Sensitivity. Cell 173, 972-988.e23. 10.1016/j.cell.2018.03.050.

19. Findlay, S., Heath, J., Luo, V.M., Malina, A., Morin, T., Coulombe, Y., Djerir, B., Li, Z., Samiei, A., Simo-Cheyou, E., et al. (2018). SHLD 2/ FAM 35A co-operates with REV 7 to coordinate DNA double-strand break repair pathway choice. The EMBO Journal 37. 10.15252/embj.2018100158.

20. Gao, S., Feng, S., Ning, S., Liu, J., Zhao, H., Xu, Y., Shang, J., Li, K., Li, Q., Guo, R., et al. (2018). An OB-fold complex controls the repair pathways for DNA double-strand breaks. Nature Communications 9, 1–10. 10.1038/s41467-018-06407-7.

21. Barazas, M., Annunziato, S., Pettitt, S.J., de Krijger, I., Ghezraoui, H., Roobol, S.J., Lutz, C., Frankum, J., Song, F.F., Brough, R., et al. (2018). The CST Complex Mediates End Protection at Double-Strand Breaks and Promotes PARP Inhibitor Sensitivity in BRCA1-Deficient Cells. Cell Rep 23, 2107–2118. 10.1016/j.celrep.2018.04.046.

22. Mirman, Z., Lottersberger, F., Takai, H., Kibe, T., Gong, Y., Takai, K., Bianchi, A., Zimmermann, M., Durocher, D., and de Lange, T. (2018). 53BP1–RIF1–shieldin counteracts DSB resection through CST- and Polα-dependent fill-in. Nature 560, 112–116. 10.1038/s41586-018-0324-7.

23. Tkáč, J., Xu, G., Adhikary, H., Young, J.T.F., Gallo, D., Escribano-Díaz, C., Krietsch, J., Orthwein, A., Munro, M., Sol, W., et al. (2016). HELB Is a Feedback Inhibitor of DNA End Resection. Molecular Cell 61, 405–418. 10.1016/j.molcel.2015.12.013.

24. Becker, J.R., Cuella-Martin, R., Barazas, M., Liu, R., Oliveira, C., Oliver, A.W., Bilham, K., Holt, A.B., Blackford, A.N., Heierhorst, J., et al. (2018). The ASCIZ-DYNLL1 axis promotes 53BP1-dependent non-homologous end joining and PARP inhibitor sensitivity. Nature Communications 9, 5406. 10.1038/s41467-018-07855-x.

25. He, Y.J., Meghani, K., Caron, M.C., Yang, C., Ronato, D.A., Bian, J., Sharma, A., Moore, J., Niraj, J., Detappe, A., et al. (2018). DYNLL1 binds to MRE11 to limit DNA end resection in BRCA1-deficient cells. Nature 563, 522–526. 10.1038/s41586-018-0670-5.

26. Olivieri, M., Cho, T., Álvarez-Quilón, A., Li, K., Schellenberg, M.J., Zimmermann, M., Hustedt, N., Rossi, S.E., Adam, S., Melo, H., et al. (2020). A Genetic Map of the Response to DNA Damage in Human Cells. Cell 182, 481-496.e21. 10.1016/j.cell.2020.05.040.

27. Francica, P., Mutlu, M., Blomen, V.A., Oliveira, C., Nowicka, Z., Trenner, A., Gerhards, N.M., Bouwman, P., Stickel, E., Hekkelman, M.L., et al. (2020). Functional Radiogenetic Profiling Implicates ERCC6L2 in Non-homologous End Joining. Cell Reports 32, 108068. 10.1016/j.celrep.2020.108068.

28. Wang, J., Aroumougame, A., Lobrich, M., Li, Y., Chen, D., Chen, J., and Gong, Z. (2014). PTIP associates with Artemis to dictate DNA repair pathway choice. Genes Dev. *28*, 2693–2698. 10.1101/gad.252478.114.

29. Drané, P., Brault, M., Cui, G., and Meghani, K. (2017). TIRR regulates 53BP1 by masking its histone methyl-lysine binding function. Nature Publishing Group, 1–37. 10.1038/nature21358.

30. Clairmont, C.S., Sarangi, P., Ponnienselvan, K., Galli, L.D., Csete, I., Moreau, L., Adelmant, G., Chowdhury, D., Marto, J.A., and D'Andrea, A.D. (2020). TRIP13 regulates DNA repair pathway choice through REV7 conformational change. Nature Cell Biology 22, 87–96. 10.1038/s41556-019-0442-y.

31. Ray Chaudhuri, A., Callen, E., Ding, X., Gogola, E., Duarte, A.A., Lee, J.-E., Wong, N., Lafarga, V., Calvo, J.A., Panzarino, N.J., et al. (2016). Replication fork stability confers chemoresistance in BRCA-deficient cells. Nature 535, 382–387. 10.1038/nature18325.

32. Ying, S., Hamdy, F.C., and Helleday, T. (2012). Mre11-dependent degradation of stalled DNA replication forks is prevented by BRCA2 and PARP1. Cancer Research 72, 2814–2821. 10.1158/0008-5472.CAN-11-3417.

33. Lemaçon, D., Jackson, J., Quinet, A., Brickner, J.R., Li, S., Yazinski, S., You, Z., Ira, G., Zou, L., Mosammaparast, N., et al. (2017). MRE11 and EXO1 nucleases degrade reversed forks and elicit MUS81-dependent fork rescue in BRCA2-deficient cells. Nature Communications 8. 10.1038/s41467-017-01180-5.

34. Schlacher, K., Christ, N., Siaud, N., Egashira, A., Wu, H., and Jasin, M. (2011). Double-strand break repair-independent role for BRCA2 in blocking stalled replication fork degradation by MRE11. Cell 145, 529–542. 10.1016/j.cell.2011.03.041.

35. Schlacher, K., Wu, H., and Jasin, M. (2012). A Distinct Replication Fork Protection Pathway Connects Fanconi Anemia Tumor Suppressors to RAD51-BRCA1/2. Cancer Cell 22, 106–116. 10.1016/j.ccr.2012.05.015.

36. Rondinelli, B., Gogola, E., Yücel, H., Duarte, A.A., Van De Ven, M., Van Der Sluijs, R., Konstantinopoulos, P.A., Jonkers, J., Ceccaldi, R., Rottenberg, S., et al. (2017). EZH2 promotes degradation of stalled replication forks by recruiting MUS81 through histone H3 trimethylation. Nature Cell Biology 19, 1371–1378. 10.1038/ncb3626.

37. Taglialatela, A., Alvarez, S., Leuzzi, G., Sannino, V., Ranjha, L., Huang, J.-W., Madubata, C., Anand, R., Levy, B., Rabadan, R., et al. (2017). Restoration of Replication Fork Stability in BRCA1- and BRCA2-Deficient Cells by Inactivation of SNF2-Family Fork Remodelers. Molecular cell 68, 414-430.e8. 10.1016/j.molcel.2017.09.036.
